# A Comprehensive Evaluation of PCR Primers to Amplify the *nifH* Gene of Nitrogenase

**DOI:** 10.1371/journal.pone.0042149

**Published:** 2012-07-25

**Authors:** John Christian Gaby, Daniel H. Buckley

**Affiliations:** Department of Crop and Soil Sciences, Cornell University, Ithaca, New York, United States of America; Catalan Institute for Water Research (ICRA), Spain

## Abstract

The *nifH* gene is the most widely sequenced marker gene used to identify nitrogen-fixing *Bacteria* and *Archaea*. Numerous PCR primers have been designed to amplify *nifH*, but a comprehensive evaluation of *nifH* PCR primers has not been performed. We performed an *in silico* analysis of the specificity and coverage of 51 universal and 35 group-specific *nifH* primers by using an aligned database of 23,847 *nifH* sequences. We found that there are 15 universal *nifH* primers that target 90% or more of nitrogen fixers, but that there are also 23 *nifH* primers that target less than 50% of *nifH* sequences. The *nifH* primers we evaluated vary in their phylogenetic bias and their ability to recover sequences from commonly sampled environments. In addition, many of these primers will amplify genes that do not mediate nitrogen fixation, and thus it would be advisable for researchers to screen their sequencing results for the presence of non-target genes before analysis. Universal primers that performed well *in silico* were tested empirically with soil samples and with genomic DNA from a phylogenetically diverse set of nitrogen-fixing strains. This analysis will be of great utility to those engaged in molecular analysis of *nifH* genes from isolates and environmental samples.

## Introduction

Nitrogen-fixing microorganisms are globally significant in that they provide the only natural biological source of fixed nitrogen in the biosphere. These organisms enzymatically transform dinitrogen gas from the atmosphere into ammonium equivalents needed for biosynthesis of essential cellular macromolecules. Nitrogen-fixing bacteria are diverse, and most of the known taxa have not yet been cultivated in the laboratory [1]. Nitrogen fixation is carried out by the nitrogenase enzyme whose multiple subunits are encoded by the genes *nifH*, *nifD*, and *nifK* (as reviewed in [2]). Of the three, *nifH* (encoding the nitrogenase reductase subunit) is the most sequenced and has become the marker gene of choice for researchers studying the phylogeny, diversity, and abundance of nitrogen-fixing microorganisms. Thus, many PCR primers have been developed to target the *nifH* gene with the purpose of amplifying this gene sequence from environmental samples.

Through use of *nifH* as a marker gene, researchers have been able to characterize aspects of the diversity and ecology of nitrogen-fixing *Bacteria* and *Archaea*. A wide range of environments have been sampled for *nifH* gene diversity including marine [3], terrestrial [4], extreme [5], anthropogenic [6], host-associated [7], and agricultural [8]. Analysis of these data indicate that the distribution of diazotrophs in the environment varies as a function of habitat type [1]. While more than 3,358 OTU_0.05_ *nifH* sequence types have been determined, the global census of diazotroph diversity remains far from complete [9]. Rates of nitrogen fixation have been associated with both *nifH* abundance [10] and *nifH* diversity [11], and thus knowledge of diazotroph community structure and dynamics is required to understand the ecological constraints on nitrogen fixation in microbial communities.

Phylogenetic analyses of *nifH* gene sequences have revealed five primary clusters of genes homologous to *nifH* [12]–[15]. Cluster I consists of aerobic nitrogen fixers including *Proteobacteria*, *Cyanobacteria*, *Frankia*, and *Paenibacillus*. Cluster II is generally thought of as the alternative nitrogenase cluster because it contains sequences from FeFe and FeV nitrogenases which differ from the conventional FeMo cofactor-containing nitrogenase. Cluster III consists of anaerobic nitrogen fixers from *Bacteria* and *Archaea* including for instance the *Desulfovibrionaceae*, *Clostridia*, *Spirochataes*, and *Methanobacteria*. Cluster IV and cluster V contain sequences that are paralogs of *nifH* and which are not involved in nitrogen fixation [13].

We set out to provide a comprehensive evaluation of primer coverage for researchers wishing to use the *nifH* gene as a molecular marker for the study of nitrogen-fixing *Bacteria* and *Archaea*. Primers that target diverse *nifH* sequences must be degenerate to encompass the sequence variability of the *nifH* gene, and Zehr and McReynolds were the first to design such degenerate primers [16], [17]. There have since been numerous efforts to design both universal and group-specific *nifH* primer sets. In a survey of the literature, we have found 51 universal and 35 group-specific primers that have been paired to make 42 universal and 19 group-specific primer sets. We have performed an *in silico* evaluation of all of these *nifH* primers using an aligned database of all publicly available *nifH* sequences which we constructed previously [9]. We then performed empirical tests of the best of these primers using genomic DNA from a phylogenetically diverse set of nitrogen fixers and DNA from soil.

## Results

**Figure 1:**
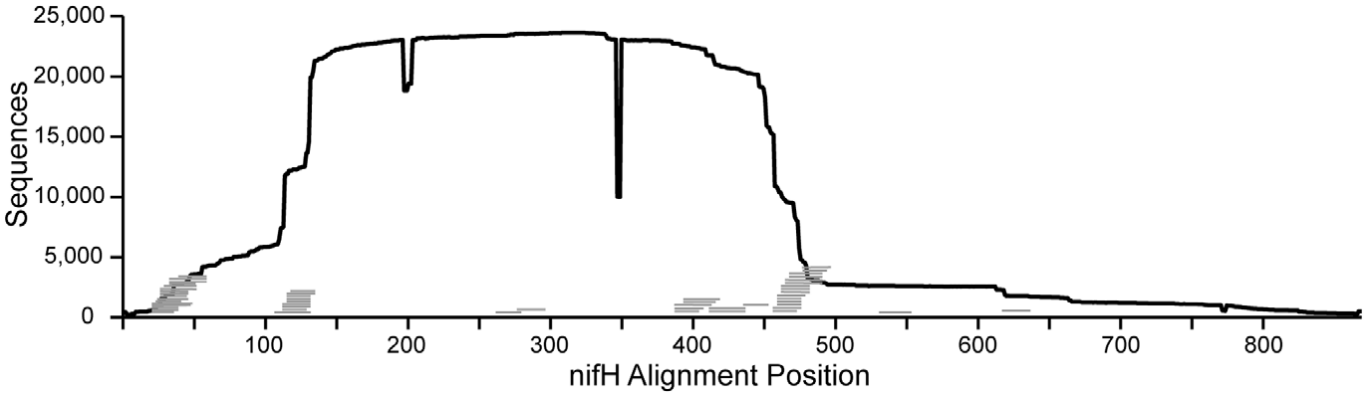
Coverage of the *nifH* gene by sequences and primers in the *nifH* database. The number of sequences in the *nifH* database is depicted in relation to alignment position along the gene. Alignment positions are referenced to the *nifH* nucleotide position from *Azotobacter vinelandii* (Genbank ACCN# M20568). Universal *nifH* primer sequences listed in Table 1 and 2 are indicated by grey horizontal lines.

Any effort to assess PCR primer coverage *in silico* must account for variation in sequence depth along the gene alignment of the database being queried. We observe that nucleotide positions near the beginning and end of the *nifH* gene alignment are under-represented in sequence databases relative to nucleotide positions in the middle of the gene alignment (Figure 1). This problem occurs because a majority of *nifH* sequences have been generated using PCR primers that bind to conserved nucleotide positions found within the *nifH* gene. A majority of the 393 full-length *nifH* sequences currently present in the *nifH* database are derived from sequenced genomes. The two dips in nucleotide coverage (at position 199 and 350 in Figure 1) result from insertions in the *Azotobacter vinelandii nifH* reference sequence relative to other genes in the alignment. In addition, some sequences in the alignment have insertions relative to *A. vinelandii* (data not shown). Due to the variations observed in sequence depth along the alignment, all estimates of primer coverage were calculated with respect to the total number of sequences available at the alignment positions where each primer binds.

We mapped the 51 universal primers to their complementary binding positions along the *A. vinelandii nifH* gene (Figure 1, Figure S1). Many primers bind to the same region (Figure 1, Figure S1), and thus may vary only slightly in binding position, oligonucleotide length, or degeneracy.

**Table 1:** Properties of universal primers and their coverage for phylogenetic and environmental groupings in the *nifH* database; continued in Table 2.

					*nifH* a (%)			Specific groupings^b^ (%)								Environ.^c^ (%)			
Primer^d^	Name	Pos.^e^	Deg.^f^	T_m_ (°C)	0	1	2	Pr	Cy	III	IA	Fr	Pb	Ep	IV	Soil	Mat	Sea	Ref.^g^
GCIWTYTAYGGNAARGG	Nh21F	19–35	64	51.8–61.9	93	96	103	89	100	100	100	100	91	83	76	78	100	100	[42]
GCIWTITAYGGNAARGGNGG	nifH19F	19–38	128	59.5–69.5	94	96	96	89	100	100	100	100	91	100	79	78	100	100	[43]
GCIWTYTAYGGIAARGGIGG	Ueda19F	19–38	16	62.4–67.9	93	96	96	89	100	100	100	100	91	83	76	78	100	100	[44]
GCIWTHTAYGGIAARGGIGGIATHGGIAA	IGK3	19–47	72	69.4–75.3	92	95	95	87	100	98	96	100	91	100	78	72	100	100	[45]
GCGTTCTACGGTAAGGGCGGTATCGGNAAR	K07-F	19–48	8	71.0–72.8	1	3	10	2	0	0	0	0	0	0	0	0	0	0	[27]
TTYTAYGGNAARGGNGG	nifH4	22–38	128	49.8–63.5	52	91	94	71	100	4	43	93	91	13	23	43	100	78	[46]
TCTACGGAAAGGGCGGTATCGG	primer-f	23–44	1	66.5	10	14	31	13	0	13	0	0	0	23	0	0	0	0	[47]
TACGGCAARGGTGGNATHG	FGPH19	25–43	24	58.2–66.2	7	24	63	9	0	4	1	71	0	2	4	3	0	0	[48]
TACGGYAARGGBGGYATCGG	IGK-Poly^h^	25–44	24	60.3–70.6	18	50	72	22	45	6	16	29	17	0	9	6	100	33	[20]
TACGG(P/K)AAKGG(P/G)GG(P/K)ATPGG	PicenoF44	25–44	8	NA	50	85	96	59	96	35	25	79	33	59	31	34	100	89	[49]
TAYGGIAARGGIGGIATYGGIAARTC	F1	25–50	4096	60.4–74.5	79	95	96	84	100	69	65	93	83	87	80	83	100	100	[18]
GGHAARGGHGGHATHGGNAARTC	MehtaF	28–50	1296	57.4–72.9	59	78	87	46	96	77	44	94	44	51	69	67	100	100	[5]
AARGGNGGNATHGGNAA^i^	IGK	31–47	384	62.1–72.5	90	98	104	91	100	93	70	95	89	94	95	95	100	100	[26]
AAAGGYGGWATCGGYAARTCCACCAC	nifHF-Rösch^h^	31–56	16	66.0–71.6	14	25	44	11	34	5	9	70	22	14	0	34	100	5	[24]
AAAGGYGGWATCCGYAARTCCACCAC	rösch F-1b^h^	31–56	16	66.0–71.6	0	14	25	0	0	0	0	0	0	0	0	0	0	0	[25]
GGTATYGGYAARTCSACSAC	RL28	37–56	32	57.7–64.1	41	70	84	43	21	41	62	57	6	12	3	24	100	5	[50]
ATHGTIGGITGYGAYCCIAARGCIGA	KAD3	106–131	16	70.1–76.8	70	84	94	89	90	15	80	97	80	82	1	70	100	24	[45]
GGNTGYGAYCCNAARGC	469	112–128	128	53.4–67.4	88	92	98	82	94	92	95	98	79	96	35	77	100	73	[20]
GGITGTGAYCCNAAVGCNGA	nif112	112–131	96	60.9–70.4	29	90	92	21	37	30	34	36	28	69	11	30	60	22	[43]
GGITGYGAYCCNAAVGCNGA	nifH-univ-f112	112–131	192	60.9–72.7	88	91	98	82	91	92	96	98	79	97	35	77	100	73	[21]
TGYGAYCCNAARGCNGA	nifH2	115–131	128	54.0–68.1	95	98	98	93	98	96	98	98	98	96	37	93	99	89	[16]
TGYGAYCCIAARGCIGA	Kadino	115–131	8	60.2–67.9	95	98	98	93	98	96	98	98	98	96	37	93	99	89	[20]
TGYGAYCCIAAIGCIGA	F2	115–131	4	62.3–67.9	96	98	98	94	98	98	99	99	98	98	37	94	99	93	[18]
TGCGAYCCSAARGCBGACTC	polF	115–134	24	63.8–70.1	39	70	88	51	12	26	41	61	34	6	3	54	15	8	[20]
GAYCCNAARGCNGACTC	nifH11	118–134	64	52.7–63.4	72	96	98	79	67	50	82	97	78	48	17	78	66	55	[51]
CTCCGGGCCRCCNGAYTC	FGPH273′	262–279	16	63.7–70.7	11	44	79	11	0	3	6	93	0	3	1	14	2	5	[48]
GMRCCIGGIGTIGGYTGYGC	nifH-2f	277–296	16	69.2–78.3	87	99	99	92	80	74	94	98	95	72	20	85	85	79	[19]
CCRCCRCANACMACGTC	Cy55Nh428R	388–404	32	56.6–67.5	66	94	99	68	76	49	63	76	55	49	23	56	65	80	[42]
AAICCRCCRCAIACIACRTC	Ueda407R	388–407	8	63.9–70.6	91	99	99	92	95	85	90	99	64	79	71	87	95	93	[44]
ATIGCRAAICCICCRCAIACIACRTC	DVV	388–413	8	71.7–75.8	94	98	99	93	94	94	96	98	90	95	52	91	96	93	[45]
GGCATNGCRAANCCVCCRCANAC	MehtaR	394–416	768	63.2–75.1	92	98	99	92	94	89	95	98	89	89	49	89	97	92	[5]

a Data indicate primer binding to all *nifH* sequences in the database with 0, 1, and 2 mismatches allowed. In some cases highly degenerate primers bind to multiple positions in the sequence generating coverage values that exceed 100%.

b Data indicate primer binding to specific groupings in the *nifH* phylogeny. Abbreviations are as follows: *Alpha, Beta, and Gamma Proteobacteria* (**Pr**); *Cyanobacteria* (**Cy**); Cluster III (**III**); Cluster IA (**IA**); *Paenibacillus* (**Pb**); *Frankia* (**Fr**); *Epsilon Proteobacteria* Containing Cluster (**Ep**); paralogous sequences in Cluster IV (**IV**).

c Primer coverage queried against sequences recovered from specific environments (Environ.) as described in methods. Environments include: soils (**Soil**), microbial mats (**Mat**), and pelagic marine samples (**Sea**).

d Sequences are given in the 5′ to 3′ direction, IUPAC characters are used, and I = Inosine.

e Position is relative to *A. vinelandii nifH* (Genbank ACCN# M20568).

f Degeneracy is given as the number of oligonucleotides that comprise the primer.

g References in which the primers are described.

h We altered these primer names in order to distinguish them from primers with similar name and sequence composition that originate from other sources.

i The 5′ linker sequence ATA GGA TCC was removed from this primer.

NA: Data not available as described in Methods.

**Table 2:** Properties of universal primers and their coverage for phylogenetic and environmental groupings in the *nifH* database; continued from Table 1.

					*nifH* a (%)			Specific groupings^b^ (%)								Environ.^c^ (%)			
Primer^d^	Name	Pos.^e^	Deg.^f^	T_m_ (°C)	0	1	2	Pr	Cy	III	IA	Fr	Pb	Ep	IV	Soil	Mat	Sea	Ref.^g^
ATIGGCATIGCRAAICCICCRCAIAC	VCG	394–419	4	73.9–76.7	93	98	99	93	94	94	95	98	90	96	33	91	96	91	[45]
TGGGCYTTGTTYTCRCGGATYGGCAT	nifHRc	412–437	16	69.1–74.2	11	34	51	18	0	0	11	0	13	0	0	12	3	9	[24]
TGSGCYTTGTCYTCRCGGATBGGCAT	nifHRb	412–437	48	70.0–76.0	0	33	56	1	0	0	0	0	0	0	0	1	0	0	[24]
SACGATGTAGATPTCCTG	PicenoR436	436–453	4	NA	34	60	83	51	10	24	13	97	7	1	3	36	28	22	[49]
TCIGGIGARATGATGGC	R6	457–473	2	61.1–62.5	96	97	99	97	99	86	99	99	102	97	15	94	99	97	[18]
ATSGCCATCATYTCRCCGGA	polR	457–476	8	63.7–67.5	35	63	86	36	15	19	40	90	23	12	0	55	7	2	[20]
ADNGCCATCATYTCNCC	nifH1	460–476	96	52.5–63.9	94	99	99	94	96	91	96	99	103	80	13	91	98	87	[16]
ADWGCCATCATYTCRCC	nifH22	460–476	24	53.2–60.9	17	89	98	16	23	21	22	1	49	20	3	11	31	36	[51]
ANDGCCATCATYTCNCC	nifH2-ZANI^i^	460–476	96	52.5–63.6	54	98	99	48	63	70	73	11	83	83	10	63	61	76	[46]
TANANNGCCATCATYTCNCC	470	460–479	512	53.8–65.7	80	82	98	84	43	62	88	99	80	98	9	79	6	95	[20]
GCRTAIABNGCCATCATYTC	nifH-univ-463r	463–482	48	55.7–63.8	85	87	88	91	50	62	91	99	88	99	8	93	83	72	[43]
GCRTAIAIIGCCATCATYTC	Emino	463–482	4	60.2–63.4	86	87	88	91	50	64	91	99	88	100	8	93	83	76	[20]
ATGATGGCSATGTAYGCSGCSAACAA	nifHR-2^i^	466–491	16	70.0–71.7	49	58	87	36	17	35	53	99	60	58	0	72	100	4	[24]
TTGTTSGCSGCRTACATSGCCATCAT	nifHR	466–491	16	70.0–71.7	49	58	87	36	17	35	53	99	60	58	0	72	100	4	[27]
TTGTTGGCIGCRTASAKIGCCAT	nifH-3r	469–491	8	68.5–72.1	48	89	94	66	7	39	72	4	30	44	3	77	100	0	[19]
ATRTTRTTNGCNGCRTA	nifH3	494–478	128	46.1–61.5	94	95	98	93	96	86	88	100	78	93	50	93	100	100	[46]
YAAATRTTRTTNGCNGCRTA	YAA-poly^i^	478–497	256	49.5–63.5	1	12	51	0	0	7	1	0	0	0	5	0	0	21	[20]
CAGATCAGVCCGCCSAGRCGMAC	RL25	532–554	24	67.5–74.1	4	29	61	5	0	8	1	0	0	0	0	1	0	0	[50]
GGCACGAAGTGGATCAGCTG	primer-r	619–638	1	64.3	4	16	43	3	0	24	0	0	0	29	0	0	0	0	[47]
GCTACTACYTCGCCSGA	AMR-R				0	0	0	0	0	0	0	0	0	0	0	0	0	0	[27]

a Data indicate primer binding to all *nifH* sequences in the database with 0, 1, and 2 mismatches allowed. In some cases highly degenerate primers bind to multiple positions in the sequence generating coverage values that exceed 100%.

b Data indicate primer binding to specific groupings in the *nifH* phylogeny. Abbreviations are as follows: *Alpha, Beta, and Gamma Proteobacteria* (**Pr**); *Cyanobacteria* (**Cy**); Cluster III (**III**); Cluster IA (**IA**); *Paenibacillus* (**Pb**); *Frankia* (**Fr**); *Epsilon Proteobacteria* Containing Cluster (**Ep**); paralogous sequences in Cluster IV (**IV**).

c Primer coverage queried against sequences recovered from specific environments (Environ.) as described in methods. Environments include: soils (**Soil**), microbial mats (**Mat**), and pelagic marine samples (**Sea**).

d Sequences are given in the 5′ to 3′ direction, IUPAC characters are used, and I = Inosine.

e Position is relative to *A. vinelandii nifH* (Genbank ACCN# M20568).

f Degeneracy is given as the number of oligonucleotides that comprise the primer.

g References in which the primers are described.

h We altered these primer names in order to distinguish them from primers with similar name and sequence composition that originate from other sources.

NA: Data not available as described in Methods.

The quality and characteristics of universal *nifH* PCR primers vary widely (Table 1 and Table 2). Of the universal primers 15 of the 51 were found to hit 90% or more of all *nifH* sequences while 23 hit less than 50% of these sequences and 9 hit 10% or fewer sequences (Table 1 and Table 2). In general, those universal primers that had >90% coverage for clusters I and III did not demonstrate systematic bias against individual phylogenetic groups within these clusters (Table 1 and Table 2). The primer KAD3 is notable, however, because it misses much of cluster III relative to cluster I (Table 1 and Table 2). Those primers with the highest coverage also tended to recognize a number of non-target sequences from cluster IV (Table 1 and Table 2).

The group-specific primers we evaluated generally show poor coverage of the phylogenetic groups they have been designed to target, except for the *Frankia*-specific primers nifH-f1-forA, nifH-f1-forB, nifH-269, and nifH-f1-rev (Table 3). The primer cyanoR targets *Cyanobacteria*, but has coverage of only 25%, and its intended pair, primer cyanoF, has a coverage of only 1% of cyanobacterial sequences (Table 3).

**Table 3:** Properties of group-specific primers and their coverage for phylogenetic and environmental groupings in the *nifH* database.

						*nifH* a (%)			Specific groupings^b^ (%)								Environ.^c^ (%)			
Primer^d^	Name	Tg.^e^	Pos.^f^	Deg.^g^	T_m_ (°C)	0	1	2	*Pr*	*Cy*	III	IA	*Fr*	*Pb*	Ep	IV	Soil	Mat	Sea	Ref.^h^
CGCIWTYTACGGIAARGGIGG	ChenBR1	BR	18–38	512	66.6–69.8	42	81	94	63	6	10	60	85	60	27	9	0	0	0	[52]
GCSTTCTACGGMAAGGGTGG	nifH-f1-forA	Fr	19–38	4	63.9–66.7	3	7	24	0	0	0	0	85	0	0	0	0	0	0	[21]
GCRTTYTACGGYAARGGSGG	nifH-a1-forA	AP	19–38	32	60.6–69.1	14	38	70	26	20	0	4	0	18	0	0	0	100	11	[21]
TACGGNAARGGSGGNATCGGCAA	nifHF	R	25–47	64	66.7–73.9	20	47	78	32	4	11	12	0	17	7	10	17	100	11	[53]
GGTATYGGYAARTGYACYAC	primer-3	RA	37–56	32	52.6–64.8	0	19	56	0	0	0	0	0	0	0	0	0	0	0	[54]
GGCAAGTCCACCACCCAGC	nifHf1	Fr	43–61	1	67.0	1	2	4	0	0	0	0	30	0	0	0	0	0	0	[55]
ATYGTCGGYTGYGAYCCSAARGC	Olsen1	AM	106–128	64	65.0–73.6	37	66	81	53	2	3	58	57	0	14	0	38	100	0	[56]
CGTAGGTTGCGACCCTAAGGCTGA	cyanoF	Cy	108–131	1	68.8	0	0	1	0	1	0	0	0	0	0	0	0	0	0	[56]
GGCTGCGATCCCAAGGCTGA	nifH-b1-forB	AB	112–131	1	68.3	1	10	32	2	0	0	1	0	0	0	0	2	0	0	[21]
GGTTGTGACCCGAAAGCTGA	nifH-g1-forB	GP	112–131	1	64.1	0	3	10	1	1	0	0	0	0	0	0	1	0	0	[21]
GGWTGTGATCCWAARGCVGA	nifH-c1-forB	AN	112–131	24	58.7–64.3	1	8	25	1	1	1	0	0	0	4	0	1	0	4	[21]
GGCTGCGATCCGAAGGCCGA	nifH-a2-forB	AP	112–131	1	70.3	1	10	33	2	0	0	0	0	23	0	0	1	0	0	[21]
GGMTGCGAYCCSAARGCSGA	nifH-a1-forB	AP	112–131	32	66.2–72.7	27	58	73	29	1	31	41	22	33	5	8	15	20	0	[21]
GGBTGYGACCCSAASGCYGA	nifH-f1-forB	Fr	112–131	48	65.9–72.9	22	48	74	15	1	9	17	91	2	3	3	22	20	0	[21]
ACCCGCCTGATCCTGCACGCCAAGG	nifHFor	MS	136–160	1	74.7	11	20	32	21	0	0	5	0	0	0	0	16	0	9	[57]
TAARGCTCAAACTACCGTAT	cylnif-F	Cs	156–175	2	56.2–57.9	1	1	3	0	3	0	0	0	0	0	0	0	0	1	[58]
GAAGGTCGGCTACCAGAACA	NIFH2F	TB	231–250	1	63.1	0	2	6	1	0	0	0	0	0	0	0	2	0	0	[59]
AAGTTGATCGAGGTGATGACG	NIFH5R	TB	306–326	1	61.6	10	22	36	21	0	0	0	0	0	0	0	18	0	7	[59]
CCGGCCTCCTCCAGGTA	nifH-269	Fr	325–341	1	64.2	3	3	3	0	0	0	0	85	0	0	0	4	0	0	[60]
ATTTAGACTTCGTTTCCTAC	cylnif-R	Cs	356–375	1	54.6	1	1	4	0	3	0	0	0	0	0	0	0	0	1	[58]
ACGATGTAGATTTCCTGGGCCTTGTT	NifHRev	MS	427–452	1	67.5	13	29	43	23	0	0	6	0	3	0	0	15	1	3	[57]
GACGATGTAGATYTCCTG	primer 4 = AQE	RA	436–453	2	53.8–55.1	24	54	81	33	8	21	12	69	7	0	1	19	24	13	[54]
GCATACATCGCCATCATTTCACC	cyanoR	Cy	460–482	1	63.6	4	8	23	2	25	0	1	0	0	0	0	1	0	8	[56]
GCGTACATSGCCATCATCTC	nifH-f1-rev	Fr	463–482	2	62.2–62.3	23	44	60	14	0	3	35	94	0	7	0	20	0	0	[21]
GCGTACATGGCCATCATCTC	nifH-b1-rev	AB	463–482	1	62.3	8	32	53	9	0	3	33	6	0	7	0	18	0	0	[21]
GCGTACATGGCCATCATCTC	nifH-g1-rev	GP	463–482	1	62.3	8	32	53	9	0	3	33	6	0	7	0	18	0	0	[21]
GCATAYASKSCCATCATYTC	nifH-c1-rev	AN	463–482	8	55.4–62.3	1	13	58	1	0	3	1	0	0	4	0	2	0	0	[21]
GCGTAGAGCGCCATCATCTC	nifH-a2-rev	AP	463–482	1	64.0	2	17	43	4	0	0	1	0	0	1	0	3	0	0	[21]
GCATAGAGCGCCATCATCTC	nifH-a1-rev	AP	463–482	1	62.0	9	16	33	17	0	1	0	0	0	0	0	2	0	0	[21]
ATGGTGTTGGCGGCRTAVAKSGCCATCAT	Olsen2	AM	466–494	24	71.5–75.3	0	32	54	0	0	0	0	0	0	0	0	0	0	0	[56]
CTCGATGACGGTCATCCGGC	nifHr	Fr	671–690	1	65.9	0	3	6	0	0	0	0	0	0	24	0	0	0	0	[55]
GGIKCRTAYTSGATIACIGTCAT	ChenBR2	BR	676–698	1024	63.6–69.1	31	67	87	40	0	0	7	71	0	0	0	39	0	0	[52]
GAAGACGATCCCGACCCCGA	FGPH750	Fr	759–778	1	66.8	0	1	1	0	0	0	0	25	0	0	0	0	0	0	[48]
AGCATGTCYTCSAGYTCNTCCA	nifHI	R	785–806	32	63.3–68.8	24	41	51	44	0	0	0	0	0	0	0	100	0	0	[53]
GGTCGGGACCTCATCCTCGA	FGPD913′	Fr	NA^i^	1	66.3	10	10	10	0	0	NA	NA	100	NA	NA	0	NA	NA	NA	[48]

a Data indicate primer binding to all *nifH* sequences in the database with 0, 1, and 2 mismatches allowed. In some cases highly degenerate primers bind to multiple positions in the sequence generating coverage values that exceed 100%.

b Data indicate primer binding to specific groupings in the *nifH* phylogeny. Abbreviations are as follows: *Alpha-, Beta-, and Gammaproteobacteria* (**Pr**); *Cyanobacteria* (**Cy**); Cluster III (**III**); Cluster IA (**IA**); *Paenibacillus* (**Pb**); *Frankia* (**Fr**); *Epsilonproteobacteria* Containing Cluster (**Ep**); paralogous sequences in Cluster IV (**IV**).

c Primer coverage queried against sequences recovered from specific environments (Environ.) as described in methods. Environments include: soils (**Soil**), microbial mats (**Mat**), and pelagic marine samples (**Sea**).

d Sequences are given in the 5′ to 3′ direction, IUPAC characters are used, and I =  Inosine.

e Abbreviations indicate the Target Group (Tg.) which the primer was intended to amplify as follows: β-*Rhizobia* (BR); *Frankia* (Fr); *Alphaproteobacteria* (AP); Symbiotic rhizobia (R); reamplification of Cluster I (RA); aerobic and microaerophilic diazotrophs (AM); *Cyanobacteria* (Cy); *Alpha- and Betaproteobacteria* (AB); *Gammaproteobacteria* (GP); alternative nitrogenase cluster (AN); designed to match multiple species of *Azospirillum*, *Burkholderia*, *Gluconoacetobacter*, *Azotobacter*, *Herbaspirillum* and *Azoarcus* (MS); species of the cyanobacterial genus *Cylindrospermopsis* (Cs); *Bradyrhizobium* sp. prevalent in truffles (TB).

f Position is relative to *A. vinelandii nifH* (Genbank ACCN# M20568).

g Degeneracy is given as the number of oligonucleotides that comprise the primer.

h References in which the primers are described.

i This binding position for this primer sequence lies beyond the stop codon of *Frankia* sp. (Genbank ACCN# M21132) and cannot be represented using the *A. vinelandii* numbering system.

NA: Data not available as described in Methods.

Given that PCR requires two primers used in combination, a useful indication of specificity must account for the coverage obtained when using specific primer pairs (Tables 4 and 5). We evaluated both primer combinations that have been reported in the literature as well as new primer combinations. As expected, the coverage obtained with primer pairs is always lower than the coverage obtained for each individual primer. We evaluated 42 universal primer pair combinations, of which 7 hit >90% of *nifH* sequences in the database, 24 hit >50%, and 6 hit 10% or less. Those primer sets which had >90% coverage are 19F/nifH3, Nh21F/nifH1, Nh21F/nifH3, IGK/nifH3, F2/R6, nifH2/R6, and nifH1/nifH2 (ie: the Zehr and McReynolds primers). The 6 primer sets which hit 10% or less of cluster I and III are Primer-f/Primer-r, FGPH19/FGPH273′, FGPH19/PolR, IGK/FGPH273′, nifHF/nifHRb, and nifHF/nifHRc. While we evaluated 19 group-specific primer combinations, very few primer sets had high coverage of the designated target groups (Table 5). The primer set ChenBR1/ChenBR2 is designed to target β-*Rhizobia* but also hits 35% of the sequences within the *Alpha-, Beta-, and Gammaproteobacteria* and 75% of *Frankia* sequences. The *Frankia*-specific primer sets nifH-f1-forA/nifH-f1-rev and nifH-f1-forB/nifH-f1-rev hit 92% and 87% of *Frankia* respectively.

**Table 4:** Properties of universal primer pairs and their coverage for phylogenetic and environmental groupings in the *nifH* database.

				Specific groupings^a^ (%)								Environ.^b^ (%)		
Primer set	Pos.^c^	Len.^d^	*nifH* e	*Pr*	*Cy*	III	IA	*Fr*	*Pb*	Ep	IV	Soil	Mat	Sea
Nh21F/Cy55Nh428R	19–404	386	67	71	98	45	74	62	73	25	13	93	100	89
Ueda19F/407R	19–407	389	86	86	100	82	100	100	80	75	48	0	100	100
NH21F/nifH1	19–476	458	91	90	100	85	100	100	100	82	1	NA	100	100
nifH19F/nifH-univ463R	19–482	464	88	86	96	76	100	100	100	100	1	NA	100	100
Ueda19F/nifH-univ463r	19–482	464	87	86	96	76	100	100	100	82	1	NA	100	100
19F/nifH3	19–494	476	92	87	96	100	100	100	100	82	32	NA	100	100
Nh21F/nifH3	19–494	476	92	87	96	100	100	100	100	82	32	NA	100	100
nifH3/nifH4	22–494	473	49	68	96	0	0	100	100	27	14	NA	100	78
Primer-f/Primer-r	23–638	616	8	10	0	9	0	0	0	39	0	NA	0	0
FGPH19/FGPH273	25–279	255	3	3	0	1	0	71	0	0	0	0	0	0
PicenoF44/PicenoR436	25–453	429	33	52	2	5	6	85	0	2	0	12	0	0
F1/R6	25–473	449	85	88	100	61	94	92	100	93	6	94	100	100
FGPH19/PolR	25–476	452	6	6	0	0	7	77	0	0	0	0	0	0
F1/nifH3r	25–491	467	51	67	8	42	87	0	75	39	3	25	100	0
MehtaF/MehtaR	28–416	389	56	44	81	73	44	88	75	43	55	52	100	95
IGK/FGPH273′	31–279	249	9	12	0	1	7	35	0	0	1	8	0	0
IGK/DVV	31–413	383	83	84	85	86	68	87	89	88	70	78	100	85
IGK/VCG	31–419	389	86	86	84	91	90	87	78	96	37	74	100	100
nifHF/nifHRb	31–437	407	0	0	0	0	0	0	0	0	0	0	0	0
nifHF/nifHRc	31–437	407	3	4	0	2	0	0	0	0	0	1	0	0
IGK/primer-4 = AQE	31–453	423	30	39	6	7	3	81	13	0	0	19	0	0
IGK/PolR	31–476	446	32	32	22	31	68	24	25	44	0	32	100	0
nifHF-Rösch/nifHR	31–491	461	26	17	34	10	42	66	50	33	0	56	100	0
IGK/YAA = nifH3	31–494	464	93	90	97	96	100	100	100	100	49	100	100	100
RL28/RL25	37–554	518	5	7	0	0	0	0	0	0	0	0	0	0
KAD3/DVV	106–413	308	66	84	83	14	81	96	64	77	1	54	100	25
KAD3/VCG	106–419	314	70	84	84	15	69	96	64	83	1	62	100	30
469/R6	112–473	362	82	79	92	69	76	98	53	96	9	61	100	80
469/nifH1	112–476	365	81	76	92	78	69	98	53	77	8	57	100	71
469/470	112–479	368	83	78	91	72	78	98	53	95	8	63	100	79
nifHFor/470	112–479	368	82	78	91	72	78	98	53	95	8	62	100	79
nif112/nifH-univ463R	112–482	371	39	28	64	53	48	31	47	73	4	46	60	64
nifB/nifHRev	112–482	371	17	8	63	21	14	20	27	13	0	12	0	64
PolF/primer-4 = AQE	115–453	339	18	26	1	13	6	40	0	0	0	24	4	2
F2/R6	115–473	359	95	95	98	84	98	98	103	97	13	92	99	91
nifH2/R6	115–473	359	94	94	98	83	97	98	103	95	13	90	99	88
nifH1/nifH2	115–476	362	92	91	96	88	94	98	104	77	11	86	99	81
PolF/PolR	115–476	362	25	30	2	11	32	59	21	3	0	51	0	0
Kadino/Emino	115–482	368	83	87	51	67	79	98	87	97	7	84	100	76
Kadino/nifH-univ-463R	115–482	368	82	86	51	65	79	98	87	96	7	84	100	72
nifH11/nifH22	118–476	359	12	15	10	7	17	1	47	6	1	8	17	22
nifH-2f/nifH-3r	277–491	215	45	63	6	31	66	3	30	33	0	74	100	0

a Data indicate primer binding to specific groupings in the *nifH* phylogeny. Abbreviations are as follows: *Alpha, Beta, and Gamma Proteobacteria* (**Pr**); *Cyanobacteria* (**Cy**); Cluster III (**III**); Cluster IA (**IA**); *Paenibacillus* (**Pb**); *Frankia* (**Fr**); *Epsilon Proteobacteria* Containing Cluster (**Ep**); paralogous sequences in Cluster IV (**IV**). In some cases highly degenerate primers bind to multiple positions in the sequence generating coverage values that exceed 100%.

b Primer coverage queried against sequences recovered from specific environments (Environ.) as described in methods. Environments include: soils (**Soil**), microbial mats (**Mat**), and pelagic marine samples (**Sea**).

c Position of amplicon in *nifH* is relative to *A. vinelandii nifH* (Genbank ACCN# M20568).

d Length expected for PCR amplicon.

e Data indicate primer binding with 0 mismatches to all *nifH* sequences in the database.

NA Data not available as nucleotide information is not available for the target group in the region of primer binding.

**Table 5:** Properties of group-specific primer pairs and their coverage for phylogenetic and environmental groups.

				Specific groupings^a^ (%)								Environ.^b^ (%)		
Primer set	Pos.^c^	Len.^d^	*nifH* e	*Pr*	*Cy*	III	IA	*Fr*	*Pb*	Ep	IV	Soil	Mat	Sea
ChenBR1/ChenBR2	18–698	681	19	35	0	0	7	75	0	0	0	NA	0	0
nifH-a1-forA/nifH-a1-rev	19–482	464	5	12	0	0	0	0	0	0	0	NA	0	0
nifH-f1-forA/nifH-f1-rev	19–482	464	3	0	0	0	0	92	0	0	0	NA	0	0
nifHF/nifHI	25–806	782	15	33	0	0	0	0	0	0	0	100	0	0
primer-3/primer-4 = AQE	37–453	417	0	0	0	0	0	0	0	0	0	0	0	0
nifHf1/nifH-269	43–341	299	1	0	0	0	0	19	0	0	0	0	0	0
nifHf1/nifHr	43–690	648	0	0	0	0	0	0	0	0	0	0	0	0
Olsen1/Olsen2	106–494	389	0	0	0	0	0	0	0	0	0	0	0	0
cyanoF/cyanoR	108–482	375	0	0	0	0	0	0	0	0	0	0	0	0
nifH-a1-forB/nifH-a1-rev	112–482	371	5	11	0	0	0	0	0	0	0	2	0	0
nifH-a2-forB/nifH-a2-rev	112–482	371	0	0	0	0	0	0	0	0	0	0	0	0
nifH-b1-forB/nifH-b1-rev	112–482	371	1	2	0	0	6	0	0	0	0	5	0	0
nifH-c1-forB/nifH-c1-rev	112–482	371	1	1	0	3	1	0	0	4	0	4	0	0
nifH-f1-forB/nifH-f1-rev	112–482	371	20	4	0	2	7	87	0	0	0	6	0	0
nifH-g1-forB/nifH-g1-rev	112–482	371	1	1	0	0	0	0	0	1	0	2	0	0
nifHFor/NifHRev	136–452	317	5	9	0	0	1	0	0	0	0	3	0	0
cylnif-F/cylnif-R	156–375	220	0	0	0	0	0	0	0	0	0	0	0	0
NIFH2F/NIFH5R	231–326	96	0	1	0	0	0	0	0	0	0	1	0	0
FGPH750/FGPD913′	759^−f^	116	0	0	0	NA	NA	0	NA	NA	0	NA	NA	NA

a Data indicate primer binding to specific groupings in the *nifH* phylogeny. Abbreviations are as follows: *Alpha, Beta, and Gamma Proteobacteria* (**Pr**); *Cyanobacteria* (**Cy**); Cluster III (**III**); Cluster IA (**IA**); *Paenibacillus* (**Pb**); *Frankia* (**Fr**); *Epsilon Proteobacteria* Containing Cluster (**Ep**); paralogous sequences in Cluster IV (**IV**). In some cases highly degenerate primers bind to multiple positions in the sequence generating coverage values that exceed 100%.

b Primer coverage queried against sequences recovered from specific environments (Environ.) as described in methods. Environments include: soils (**Soil**), microbial mats (**Mat**), and pelagic marine samples (**Sea**).

c Position of amplicon in *nifH* is relative to *A. vinelandii nifH* (Genbank ACCN# M20568).

d Length expected for PCR amplicon.

e Data indicate primer binding with 0 mismatches to all *nifH* sequences in the database.

f This binding position for the reverse primer sequence lies beyond the stop codon of *Frankia* sp. (Genbank ACCN# M21132) and cannot be represented using the *A. vinelandii* numbering system.

NA Data not available as nucleotide information is not available for the target group in the region of primer binding.

Primer sets with high *in silico* coverage were used for empirical tests. When tested with DNA from soil, the primer combinations nifH2/R6, nH21f/nifH, nifH1/nifH2, Ueda19f/univ463r, and nifH3/nH21f all produced PCR products of indiscriminate size producing smeared bands in gel electrophoresis and also produced an amplified product from *E. coli* indicating a lack of specificity for *nifH* under the amplification conditions tested (Table 6, Figures S9, S10, S11, S12, S13, S14, S15). The primer combinations F2/R6, IGK3/DVV, and Ueda 19F/388R produced a band of the expected size for a diverse range of genomic and soil DNA templates (Table 6, Figures S3, S4, S5, S6, S7, S8), though Ueda 19F/388R was observed to produce an amplified product from *E. coli* indicating a lack of specificity for *nifH* under the amplification conditions tested. Overall, the primer pair IGK3/DVV produced the best performance in our empirical analysis, producing PCR products of the expected size from all nitrogen-fixing strains and soil DNA samples tested, while not generating PCR product from the negative controls or producing non-specific PCR products (Table 6, Figures S5 and S6).

**Table 6:** Empirical results of PCR using different *nifH* primer sets with DNA from isolates and soils^a^.

	AT (°C)^b^	Dv	Gu	Av	Fs	Ml	Kp	Xa	Rs	Rl	Pn	Ec	AS	LS	NT
F2/R6	51	−	+	+	−	+	+	−	ns	−	−	−	+	+	−
IGK3/DVV	58	+	+	+	+	+	+	+	+	+	+	−	+	+	−
Ueda19F/388R	51	+	+	+	−	+	+	+	+	ns	+	ns	+	+	−
nifH2/R6	44	ns	+	+	+	+	+	ns	−	+	−	ns	s	s	−
nH21f/nifH1	46	ns	Ns	+	−							ns	s	s	−
nifH1/nifH2	46	ns	+	+	−							ns	s	s	−
Ueda19f/univ463r	46	+	ns	+	−	+	+	+	−	+	+	ns	s	s	−
nifH3/nH21f	41	ns	ns	+	−							ns	s	s	−

a DNA samples and their phylogenetic affiliation in the *nifH* phylogeny from Figure S2 are: *Desulfovibrio vulgaris* Hildenborough (**Dv**), cluster III; *Geobacter uraniireducens* Rf4 (**Gu**), subcluster IA; A*zotobacter vinelandii* DJ (**Av**), *Alpha-, Beta- and Gamma-Proteobacteria; Frankia* sp. CcI3 (**Fs**), *Frankia*; *Mastigocladus laminosus* UTEX LB 1931 (**Ml**), *Cyanobacteria*; *Klebsiella pneumoniae* 342 (**Kp**), *Alpha-, Beta- and Gammaproteobacteria*; *Xanthobacter autotrophicus* Py2 (**Xa**), *Alpha-, Beta- and Gammaproteobacteria*; *Rhodobacter sphaeroides* 2.4.1 (**Rs**), *Alpha-, Beta- and Gammaproteobacteria*; *Rhizobium leguminosarium* bv. trifolii (**Rl**), *Alpha-, Beta- and Gammaproteobacteria; Polaromonas naphthalenivorans* CJ2 (**Pn**), *Alpha-, Beta- and Gammaproteobacteria*; *Eschericia coli* (**Ec**), genomic-DNA negative control; agricultural soil (**AS**); lawn soil (**LS**); No Template Control (**NT**). The symbols used are: product of the correct size (**+**), no product produced (**−**), non-specific amplification producing multiple bands or a single band of the wrong size (ns), a smeared band of indiscriminate size overlapping in size with the expected product (**s**). Blank cells indicate that the evaluation was not performed.

b Annealing Temperature (**AT**) used in PCR.

## Discussion

We report a comprehensive evaluation of *nifH* PCR primers. Our analysis of *nifH* primers reveals disparities in their sequence coverage. Variation in coverage is especially notable for primers designed to be universal, where 23 out of 51 target fewer than 50% of known *nifH* sequences and only 15 target more than 90% of sequences (Table 1). There could be several reasons for the disparity in primer coverage and specificity. Adequate primer design requires use of a sequence database representing the entire sequence diversity to be targeted by the primer. The number of sequences available in public databases has grown dramatically in recent years and earlier efforts at primer design were constrained in the past by the limited number and diversity of *nifH* sequences available. There is also a reasonable tendency to seek minimally degenerate primers due to undesirable effects that high levels of primer degeneracy can have on PCR performance. Decisions to lower degeneracy, however, could come at the cost of adequate coverage of target sequences.

Our efforts to evaluate universal *nifH* primers expand upon previous work to design universal primers for this gene. Marusina *et al.* designed *nifH* primers based upon a diverse set of *nifH* sequences and tested the resulting primers against DNA from cultivated strains [18]. The F2/R6 primer set they designed was one of the best performing in our comparison (Tables 4 and 6, Figures S3 and S4). Fedorov *et al.* later reexamined some of the primers of Marusina *et al.* because they found that primer R6 contained mismatches to certain methylobacterial *nifH* sequences, and they sought to design primers that included this group [19]. The coverage of their new primer, nifH-3r, however, is considerably lower than that of the original R6 primer matching 48% and 96% of *nifH* sequences respectively (Table 1). Poly *et al.* also designed a universal primer set, PolF/PolR, and showed that it amplified 19 of 19 test strains and worked well in soils [20]. However, the test strains they used consisted of *Alpha-, Beta-, and Gammaproteobacteria*, *Firmicutes* and *Actinobacteria* and did not include cluster IA, *Cyanobacteria*, or cluster III sequences. We found that the PolF/PolR primer set only encompassed 25% of *nifH* diversity in our database (Table 4).

By mapping the 51 universal primers to their complementary binding positions along the *A. vinelandii nifH* gene (Figure 1), it is evident that the majority of the primers correspond to conserved regions of the *nifH* gene that encode essential functions like the P-loop, Switch I, and Switch II (Figure 1; [22] ). Sequence coverage is high in regions of universal primer binding (Figure 1), and the shape of the coverage profile suggests that primer sequences have not been trimmed from a large number of sequences. If this is indeed the case, then there could be some bias in our results since the sequence fidelity between primer and target can vary as a function of the specificity of PCR conditions. If primer sequences have replaced existing *nifH* polymorphism in database sequences, then the net result would be a bias towards overestimating primer coverage. This is a common problem in public sequence databases and illustrates the need for depositors to remove primer sequences prior to sequence deposition.

Some of the primer sequences we evaluated have unusually low coverage perhaps indicating that the published sequences contain errors, a phenomenon which is not that uncommon as it has been noted in another review of primer sequences [23]. In particular, there appear to be errors in the sequences published for the primers YAA-poly, nifHRb, and röschF-1b [20], [24], [25]. In the case of primer YAA-poly it appears that the first part of the primer name “YAA” was appended to the 5′ end of the primer sequence in [20] because the original YAA primer sequence does not have these nucleotides [26]. The coverage values for the original YAA primer (the one without the 5′ YAA nucleotides) are actually those of the primer nifH3 (Table 2). For primers nifHRb and röschF-1b there appear to be single base pair errors in the primer sequences. If a single base pair mismatch is allowed for these primers it causes coverage to increase substantially (Table 1, Table 2). The primer röschF-1b [25] differs from the primer nifHF-Rösch [24] in that a G rather than a C is present at the 13th nucleotide from the 5′ terminus. In addition, the primer AMR-R, though reported as a *nifH* primer [27], does not match *nifH* and thus appears to be erroneous.

We evaluate primer coverage *in silico* but it is important to point out that universal *nifH* PCR primers have been used under a wide range of reaction conditions and variation in annealing temperatures and cycle parameters will have dramatic impacts on actual primer performance. Lowering of PCR annealing temperature, for example, lowers reaction specificity and may permit amplification of templates with mismatches in the primer binding region. Notably, for many primer sets either a nested, touchdown, or stepdown PCR approach was needed to achieve amplification of *nifH* genes from environmental samples (e.g. [28], [29] ). In Tables 1–3 we indicate primer coverage with up to two mismatches to provide an indication of the potential effects that reducing reaction stringency may have on primer performance. In addition, there are several other factors which could impact the specificity and coverage realized using PCR primers at the bench relative to predictions made using sequence databases. These factors include primer dimerization [30], hairpin formation [31], GC content [32], the location of mismatches [33], and the thermodynamics of primer binding to template [34]. For example, mismatches at the 3′ end of a primer may have a greater impact on specificity than those at the 5′ end [33] and some methods of primer design exploit this tendency in order to increase primer coverage [35]. Thus, the real test of primer performance comes at the bench. We performed empirical assessment of coverage for primers which we found targeted 90% or more of sequences in the *nifH* database. The primer combinations F2/R6, IGK3/DVV, and Ueda 19F/388R performed well with DNA from a diversity of phylogenetic groups and from soil, with IGK3/DVV performing best of all. In contrast, the primer sets Ueda19f/univ463r and nifH1/nifH2 (ie: the Zehr-McReynolds primers) had mediocre performance with soils, producing smeared bands indicative of non-specific amplification, and producing a PCR product from negative controls (Table 6, Figures S13 and S14). All other primer combinations tested had drawbacks such as poor or no soil amplification and amplification of negative controls (Table 6, Figures S9, S10, S11, S12 and S15).

There are several limitations to our approach which must be considered. First, only a few full-length *nifH* sequences are currently available and this lowers the sequence diversity represented along the termini of the *nifH* gene (Figure 1). Hence, evaluation of primers that bind near the beginning or end of the alignment must be interpreted with care, especially for phylogenetic groups that are underrepresented in sequence databases. Likewise, *nifH* diversity remains poorly characterized in some and thus estimates primer performance in specific environments must also be interpreted with care when the number of sequences from those environments are small. We refer the reader to the supplementary material (Dataset S1) which provides the number of sequences currently available for each phylogenetic group and for each environment queried. As the number of sequenced genomes increases, full length *nifH* sequences from more diverse nitrogen fixers will become available aiding future efforts at primer design and analysis. Secondly, we have made no effort to assess coverage for nested and semi-nested reactions, which are common approaches. Nested amplification strategies, when coupled with low stringency reaction conditions, can allow investigators to amplify a wider diversity of templates than would be predicted through *in silico* analysis. Logically, however, *in silico* results from nested designs would always produce a reduction in coverage relative to a single primer set design.

Some of the universal *nifH* primers amplify paralogous genes not involved in nitrogen-fixation, for example cluster IV genes (Table 1 and Table 2). The *nifH* gene shares conserved regions with genes of cluster IV and cluster V which is involved in bacteriochlorophyll synthesis [13], [22]. We find that a substantial number of *nifH* universal primers will amplify cluster IV sequences (Table 1 and Table 2). It would therefore be wise for researchers interested in assessing the diversity and phylogeny of nitrogen-fixation genes from the environment to screen their sequences for the presence of cluster IV and cluster V genes prior to OTU clustering.

Our work outlines a comprehensive approach to primer evaluation. Molecular-based studies are dependent on the effectiveness of the primer sets used to generate the sequence data which serves as our window to the microbial world. These results show that many supposedly universal primer sets miss significant portions of known *nifH* diversity. Several of the primers that performed well *in silico* were tested empirically against genomic DNA from a phylogenetically diverse set of strains. The primers that performed well both *in silico* and empirically should have the greatest utility in further studies of the *nifH* gene diversity in environmental samples.

## Materials and Methods

Primer coverage analyses were performed using an updated version of our previously described *nifH* database [9]. The current version of the database contains 23,847 sequences, representing all *nifH* sequences available in Genbank as of July 14, 2010. The database was constructed using the ARB software package [36] as described in [9]. Alignment positions are numbered relative to the *Azotobacter vinelandii* gene sequence (Genbank ACCN# M20568). The environmental origins of sequences (Tables 1–5) were determined by keyword searches of the sequence records in the *nifH* database using ARB as described in [9]. The phylogenetic trees and sequence configurations for the environmental groups may be examined as part of the ARB *nifH* database used for this work which is available at http://www.css.cornell.edu/faculty/buckley/nifH_database_2010_07_14. arb. The phylogenetic groups evaluated (Table 1–5) are labeled on the phylogenetic tree of Figure S2 which corresponds to the tree in the ARB database.

We visualized the nucleotide representation of *nifH* sequence fragments within our *nifH* database relative to the *A. vinelandii nifH* sequence (Figure 1) by first exporting in FASTA format all *nifH* sequences from the ARB database using the *A. vinlandii nifH* sequence as a filter so that only positions in the alignment where *A. vinelandii nifH* had a nucleotide were exported. The FASTA file was then opened in BioEdit [37] where we could calculate a positional nucleotide numerical summary, and the total number of sequences containing sequence information was then plotted for each position in the alignment (Figure 1).

Primer coverage calculations were performed using the EMBOSS programs fuzznuc, dreg, and primersearch [38] to analyze sequence alignment data exported in FASTA format from our *nifH* database. The program fuzznuc calculates the number of sequences in a given alignment hit by a given primer. Mismatches, or fuzzy searches, are allowed by the program and were performed with the *nifH* evaluations (Table 1–3). The program primersearch was used for the evaluation of primer pairs (Tables 4 and 5). The program dreg was used to determine the number of records in an alignment that contained sequence data in the alignment region targeted by each primer or primer pair (Tables 1–5). However, because dreg eliminates the gap characters from the FASTA alignment file from ARB, the flanking gap characters were converted to the IUPAC character S, which is preserved by dreg, and the intervening gap characters were subsequently converted to the IUPAC character N. This allowed the original column positions from the ARB alignment to be maintained and reported as output from dreg. To calculate primer and primer pair coverage, the number of hits obtained from fuzznuc or primersearch were divided by the total number of sequences with nucleotide representation in the target region(s) as indicated by dreg.

Unix bash shell scripts were employed to increase the throughput of the *in silico* primer evaluations by automating the input of multiple primer sequences and other evaluation parameters into the EMBOSS programs. The scripts were also used to parse the output files and organize the data into tables. These scripts, which would be useful for similar evaluations using databases for other functional genes, are available as supplementary material online (Text S1, S2, S3).

Primer annealing temperatures were calculated with SciTools Oligoanalyzer version 3.1 which calculates oligonucleotide melting temperatures based on nearest neighbor thermodynamics [39]. Oligoanalyzer can account for Inosine but not for P or K bases and thus melting temperatures were not calculated for PicenoF44 and PicenoR436 (Table 1). The parameters used for the calculations were 0.25 µM oligonucleotides, 50 mM Na^+^, 1.5 mM Mg^++^, and 0 mM dNTPs.

Genomic DNA was extracted from cultures of the bacterial strains listed in Table 6 according to a standard enzymatic, phenol-chloroform extraction protocol [40]. DNA concentration was determined with a Nanodrop model 1000 (Thermo Fischer Scientific, Wilmington, DE), and DNA was diluted to 1 ng µl^−1^ prior to PCR. Soil DNA was obtained from a long-term agricultural site at the William H. Miner institute, Chazy, NY described previously [41]. The agricultural soil sample comes from a tilled site used to grow corn for more than 30 years while the lawn soil sample is from a non-cultivated control site that is adjacent to the agricultural site and contains a mixed community of perennial grasses (Table 6). Soil samples were obtained by coring at 0–5 cm depth. Soil samples were sieved to 2 mm, frozen in the field using liquid nitrogen, and stored at −80°C. DNA was extracted from soils using the PowerSoil DNA Isolation Kit (MoBio, Carlsbad, CA).

Primers were synthesized and desalted by Integrated DNA technologies. All PCR reaction volumes were 50 µL with the following final reagent concentrations: 1X PCR Gold Buffer (ABI, Foster City, CA), 2.5 mM MgCl_2_ solution (ABI, Foster City, CA), 0.05% BSA (NEB, Ipswich, MA), 0.2 mM dNTPs, 1 µM each primer, 2.5 U Amplitaq Gold DNA polymerase (ABI, Foster City, CA). As template, 1 ng of genomic DNA was added, or 1 µl of soil DNA extract. To visualize the PCR products, 10 µL of the reactions were loaded onto a 50 ml, 1% agarose gel with 1 µL of SYBR Safe dye (Molecular Probes, Eugene, OR). 5 µl of Hyperladder I (Bioline, Taunton, MA) was loaded onto each gel as a molecular weight marker. Gels ran for 45 minutes at 100 volts and 500 miliamps and were then visualized and photographed. Photos of the electrophoresis gels are available as supplementary material online (Figures S3, S4, S5, S6, S7, S8, S9, S10, S11, S12, S13, S14, S15).

## Supporting Information

Figure S1 **Universal** ***nifH*** **primer map.** Universal *nifH* primers (grey lines with names) are mapped onto the sequence of *Azotobacter vinelandii* (Genbank ACCN# M20568).
(EPS)

Figure S2 **Phylogenetic tree of** ***nifH*** **sequences in the database.** The principle groups from Tables 1–5 are labeled.
(EPS)

Figure S3 **F2/R6 primer pair at 51°C annealing temperature.** Gel image of PCR products generated using the primers indicated with a range of different DNA templates. Results are summarized and full strain names are reported in Table 6. The gel images have been inverted from black to white.
(TIF)

Figure S4 **F2/R6 primer pair at 51°C annealing temperature.** Gel image of PCR products generated using the primers indicated with a range of different DNA templates. Results are summarized and full strain names are reported in Table 6. The gel images have been inverted from black to white.
(TIF)

Figure S5 **IGK3/DVV primer pair at 58°C annealing temperature.** Gel image of PCR products generated using the primers indicated with a range of different DNA templates. Results are summarized and full strain names are reported in Table 6. The gel images have been inverted from black to white.
(TIF)

Figure S6 **IGK3/DVV primer pair at 58°C annealing temperature.** Gel image of PCR products generated using the primers indicated with a range of different DNA templates. Results are summarized and full strain names are reported in Table 6. The gel images have been inverted from black to white.
(TIF)

Figure S7 **Ueda19F/388R primer pair at 51°C annealing temperature.** Gel image of PCR products generated using the primers indicated with a range of different DNA templates. Results are summarized and full strain names are reported in Table 6. The gel images have been inverted from black to white.
(TIF)

Figure S8 **Ueda19F/388R primer pair at 51°C annealing temperature.** Gel image of PCR products generated using the primers indicated with a range of different DNA templates. Results are summarized and full strain names are reported in Table 6. The gel images have been inverted from black to white.
(TIF)

Figure S9 **nifH2/R6 primer pair at 44°C annealing temperature.** Gel image of PCR products generated using the primers indicated with a range of different DNA templates. Results are summarized and full strain names are reported in Table 6. The gel images have been inverted from black to white.
(TIF)

Figure S10 **nifH2/R6 primer pair at 44°C annealing temperature.** Gel image of PCR products generated using the primers indicated with a range of different DNA templates. Results are summarized and full strain names are reported in Table 6. The gel images have been inverted from black to white.
(TIF)

Figure S11 **nH21f/nifH1 primer pair at 46°C annealing temperature.** Gel image of PCR products generated using the primers indicated with a range of different DNA templates. Results are summarized and full strain names are reported in Table 6. The gel images have been inverted from black to white.
(TIF)

Figure S12 **nifH1/nifH2 primer pair at 46°C annealing temperature.** Gel image of PCR products generated using the primers indicated with a range of different DNA templates. Results are summarized and full strain names are reported in Table 6. The gel images have been inverted from black to white.
(TIF)

Figure S13 **Ueda19f/univ463r primer pair at 46°C annealing temperature.** Gel image of PCR products generated using the primers indicated with a range of different DNA templates. Results are summarized and full strain names are reported in Table 6. The gel images have been inverted from black to white.
(TIF)

Figure S14 **Ueda19f/univ463r primer pair at 46°C annealing temperature.** Gel image of PCR products generated using the primers indicated with a range of different DNA templates. Results are summarized and full strain names are reported in Table 6. The gel images have been inverted from black to white.
(TIF)

Figure S15 **nifH3/nH21f primer pair at 41°C annealing temperature.** Gel image of PCR products generated using the primers indicated with a range of different DNA templates. Results are summarized and full strain names are reported in Table 6. The gel images have been inverted from black to white.
(TIF)

Dataset S1 (XLS)

Text S1 (TXT)

Text S2 (TXT)

Text S3 (TXT)
