# Correction: A Comprehensive Evaluation of PCR Primers to Amplify the *nifH* Gene of Nitrogenase

**DOI:** 10.1371/journal.pone.0093883

**Published:** 2014-03-28

**Authors:** The PLOS ONE Staff

The sequence for primer R6 given in Table 2 is incorrect. Please see the correct Table 2 below.

**Table 2:** Properties of universal primers and their coverage for phylogenetic and environmental groupings in the nifH database; continued from Table 1.

					*nifH* a (%)			Specific groupings b (%)								Environ. c (%)			
Primer d	Name	Pos. e	Deg. f	T_m_ (°C)	0	1	2	Pr	Cy	III	IA	Fr	Pb	Ep	IV	Soil	Mat	Sea	Ref. g
ATIGGCATIGCRAAICCICCRCAIAC	VCG	394-419	4)	73.9-76.7	93	98	99	93	94	94	95	98	90	96	33	91	96	91	[45]
TGGGCYTTGTTYTCRCGGATYGGCAT	nifHRc	412-437	16	69.1-74.2	11	34	51	18	0	0	11	0	13	0	0	12	3	9	[24]
TGSGCYTTGTCYTCRCGGATBGGCAT	nifHRb	412-437	48	70.0-76.0	0	33	56	1	0	0	0	0	0	0	0	1	0	0	[24]
SACGATGTAGATPTCCTG	PicenoR436	436-453	4	NA	34	60	83	51	10	24	13	97	7	1	3	36	28	22	[49]
GCC ATC ATY TCI CCI GA	R6	457-473	2	61.1-62.5	96	97	99	97	99	86	99	99	102	97	15	94	99	97	[18]
ATSGCCATCATYTCRCCGGA	polR	457-476	8	63.7-67.5	35	63	86	36	15	19	40	90	23	12	0	55	7	2	[20]
ADNGCCATCATYTCNCC	nifH1	460-476	96	52.5-63.9	94	99	99	94	96	91	96	99	103	80	13	91	98	87	[16]
ADWGCCATCATYTCRCC	nifH22	460-476	24	53.2-60.9	17	89	98	16	23	21	22	1	49	20	3	11	31	36	[51]
ANDGCCATCATYTCNCC	nifH2-ZANI^ i^	460-476	96	52.5-63.6	54	98	99	48	63	70	73	11	83	83	10	63	61	76	[46]
TANANNGCCATCATYTCNCC	470	460-479	512	53.8-65.7	80	82	98	84	43	62	88	99	80	98	9	79	6	95	[20]
GCRTAIABNGCCATCATYTC	nifH-univ-463r	463-482	48	55.7-63.8	85	87	88	91	50	62	91	99	88	99	8	93	83	72	[43]
GCRTAIAIIGCCATCATYTC	Emino	463-482	4	60.2-63.4	86	87	88	91	50	64	91	99	88	100	8	93	83	76	[20]
ATGATGGCSATGTAYGCSGCSAACAA	nifHR-2^ i^	466-491	16	70.0-71.7	49	58	87	36	17	35	53	99	60	58	0	72	100	4	[24]
TTGTTSGCSGCRTACATSGCCATCAT	nifHR	466-491	16	70.0-71.7	49	58	87	36	17	35	53	99	60	58	0	72	100	4	[27]
TTGTTGGCIGCRTASAKIGCCAT	nifH-3r	469-491	8	68.5-72.1	48	89	94	66	7	39	72	4	30	44	3	77	100	0	[19]
ATRTTRTTNGCNGCRTA	nifH3	494-478	128	46.1-61.5	94	95	98	93	96	86	88	100	78	93	50	93	100	100	[46]
YAAATRTTRTTNGCNGCRTA	YAA-poly^i^	478-497	256	49.5-63.5	1	12	51	0	0	7	1	0	0	0	5	0	0	21	[20]
CAGATCAGVCCGCCSAGRCGMAC	RL25	532-554	24	67.5-74.1	4	29	61	5	0	8	1	0	0	0	0	1	0	0	[50]
GGCACGAAGTGGATCAGCTG	primer-r	619-638	1	64.3	4	16	43	3	0	24	0	0	0	29	0	0	0	0	[47]
GCTACTACYTCGCCSGA	AMR-R				0	0	0	0	0	0	0	0	0	0	0	0	0	0	[27]

a Data indicate primer binding to all *nifH* sequences in the database with 0, 1, and 2 mismatches allowed. In some cases highly degenerate primers bind to multiple positions in the sequence generating coverage values that exceed 100%.

b Data indicate primer binding to specific groupings in the *nifH* phylogeny. Abbreviations are as follows: *Alpha, Beta, and Gamma Proteobacteria* (**Pr**); *Cyanobacteria* (**Cy**)**;** Cluster III (**III**); Cluster IA (**IA**); *Paenibacillus* (**Pb**); *Frankia* (**Fr**); *Epsilon Proteobacteria* Containing Cluster (**Ep**); paralogous sequences in Cluster IV (**IV**).

c Primer coverage queried against sequences recovered from specific environments (Environ.) as described in methods. Environments include: soils (**Soil**), microbial mats (**Mat**), and pelagic marine samples (**Sea**).

d Sequences are given in the 5' to 3' direction, IUPAC characters are used, and I = Inosine.

e Position is relative to *A. vinelandii nifH* (Genbank ACCN# M20568).

f Degeneracy is given as the number of oligonucleotides that comprise the primer.

g References in which the primers are described.

h We altered these primer names in order to distinguish them from primers with similar name and sequence composition that originate from other sources.

NA: Data not available as described in Methods.
