# Supplementary figures and images for: A Comprehensive Evaluation of PCR Primers to Amplify the nifH Gene of Nitrogenase
Source: PLoS One. 2012 Jul 25;7(7):e42149. doi: 10.1371/journal.pone.0042149 (PMC3405036; doi:10.1371/journal.pone.0042149)

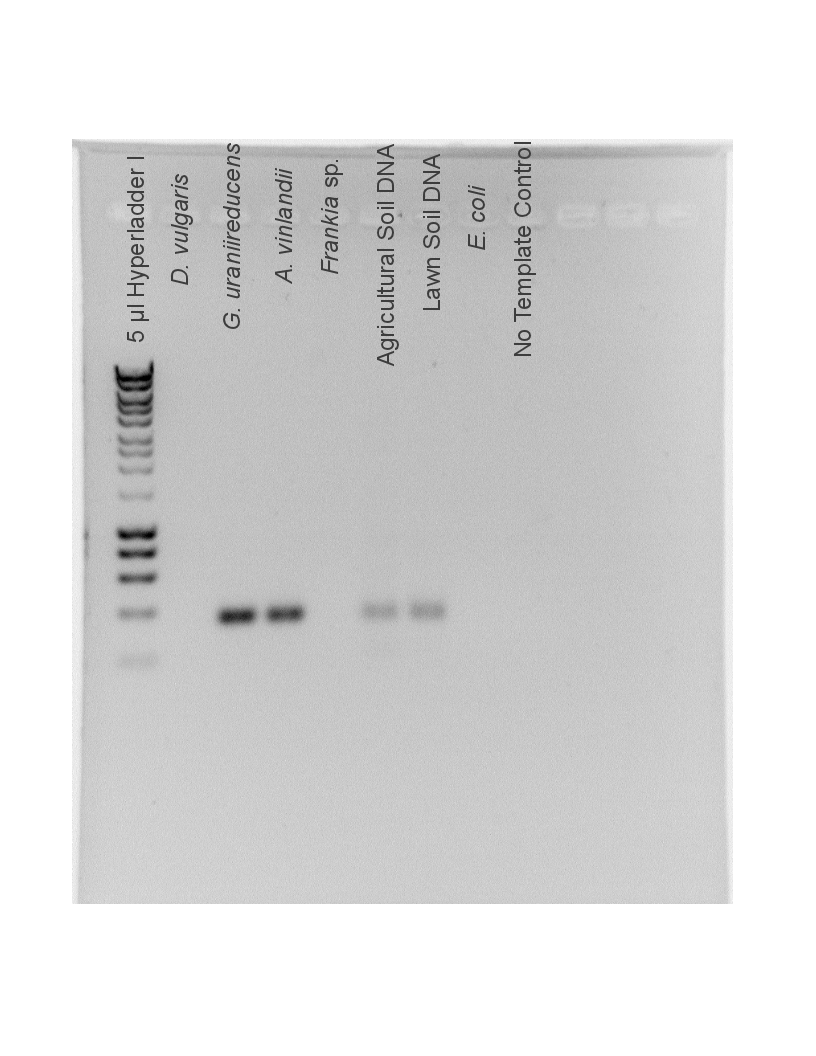

Supplement: Figure S3 — F2/R6 primer pair at 51°C annealing temperature. Gel image of PCR products generated using the primers indicated with a range of different DNA templates. Results are summarized and full strain names are reported in Table 6. The gel images have been inverted from black to white. (TIF) [file pone.0042149.s003.tif]

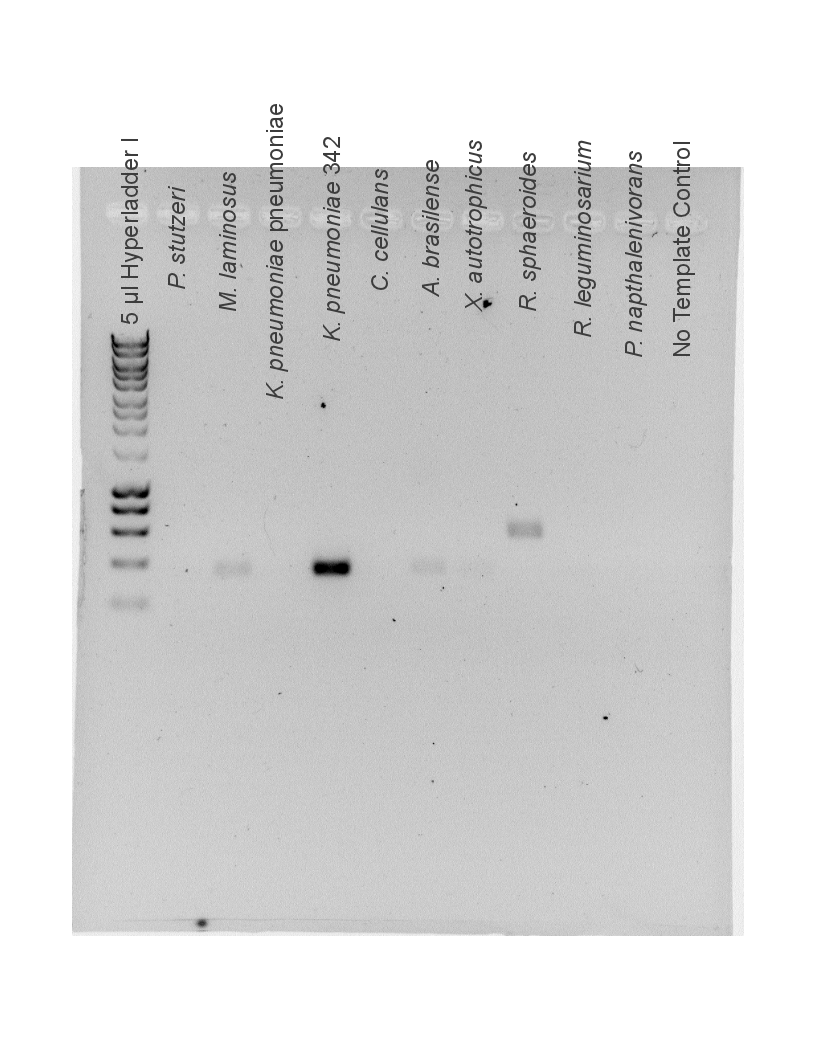

Supplement: Figure S4 — F2/R6 primer pair at 51°C annealing temperature. Gel image of PCR products generated using the primers indicated with a range of different DNA templates. Results are summarized and full strain names are reported in Table 6. The gel images have been inverted from black to white. (TIF) [file pone.0042149.s004.tif]

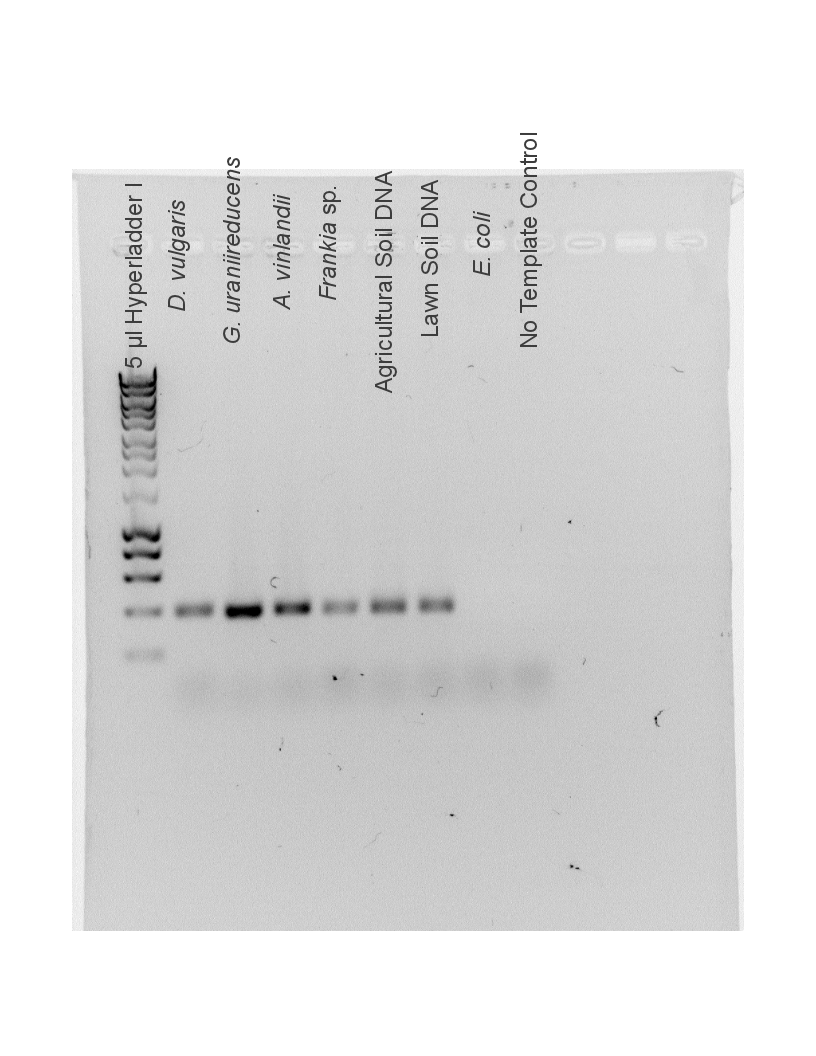

Supplement: Figure S5 — IGK3/DVV primer pair at 58°C annealing temperature. Gel image of PCR products generated using the primers indicated with a range of different DNA templates. Results are summarized and full strain names are reported in Table 6. The gel images have been inverted from black to white. (TIF) [file pone.0042149.s005.tif]

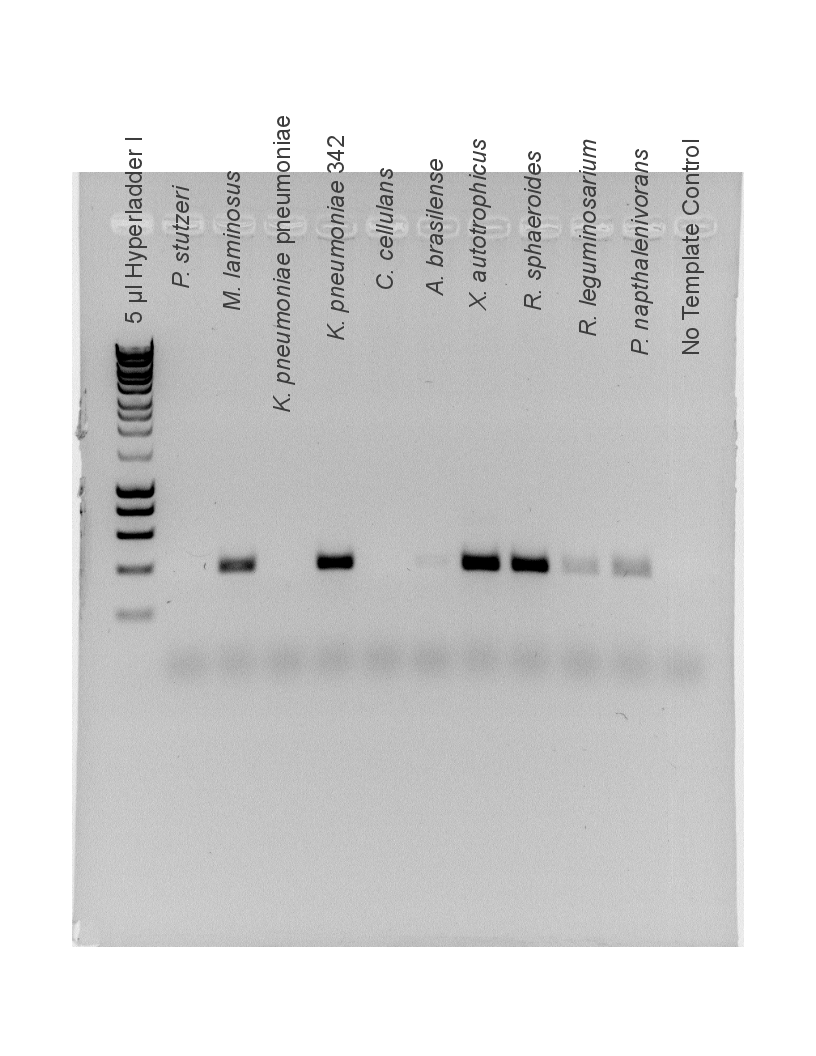

Supplement: Figure S6 — IGK3/DVV primer pair at 58°C annealing temperature. Gel image of PCR products generated using the primers indicated with a range of different DNA templates. Results are summarized and full strain names are reported in Table 6. The gel images have been inverted from black to white. (TIF) [file pone.0042149.s006.tif]

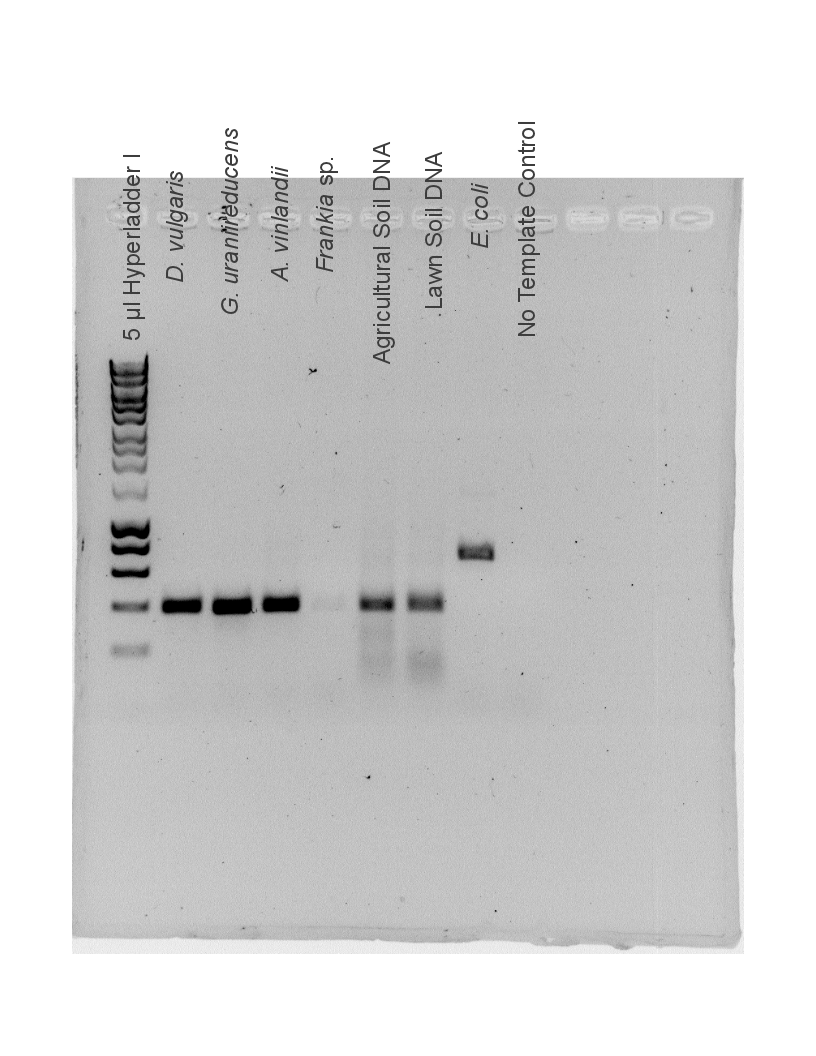

Supplement: Figure S7 — Ueda19F/388R primer pair at 51°C annealing temperature. Gel image of PCR products generated using the primers indicated with a range of different DNA templates. Results are summarized and full strain names are reported in Table 6. The gel images have been inverted from black to white. (TIF) [file pone.0042149.s007.tif]

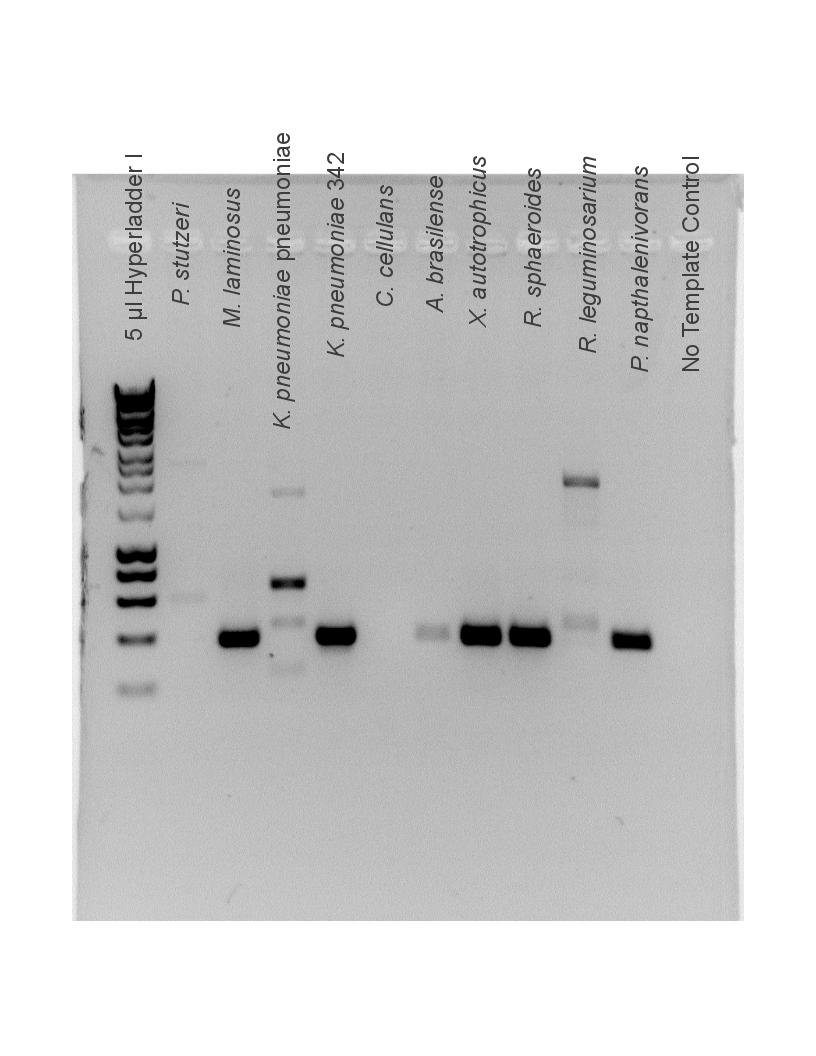

Supplement: Figure S8 — Ueda19F/388R primer pair at 51°C annealing temperature. Gel image of PCR products generated using the primers indicated with a range of different DNA templates. Results are summarized and full strain names are reported in Table 6. The gel images have been inverted from black to white. (TIF) [file pone.0042149.s008.tif]

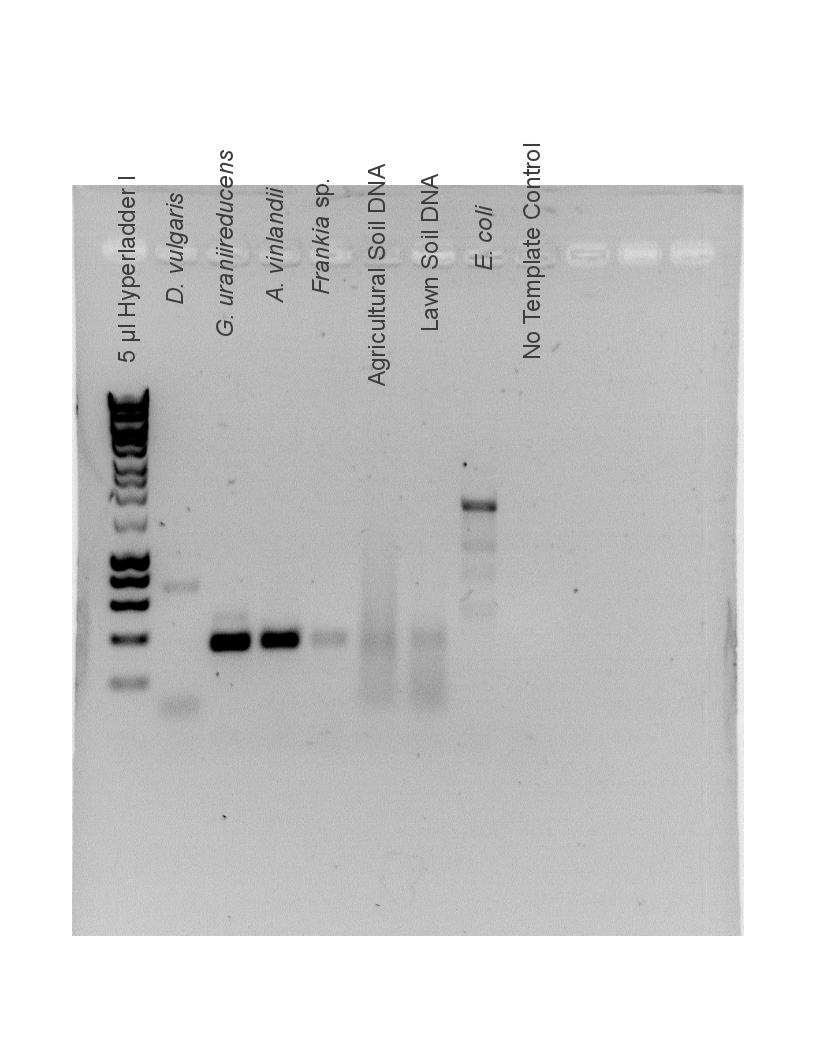

Supplement: Figure S9 — nifH2/R6 primer pair at 44°C annealing temperature. Gel image of PCR products generated using the primers indicated with a range of different DNA templates. Results are summarized and full strain names are reported in Table 6. The gel images have been inverted from black to white. (TIF) [file pone.0042149.s009.tif]

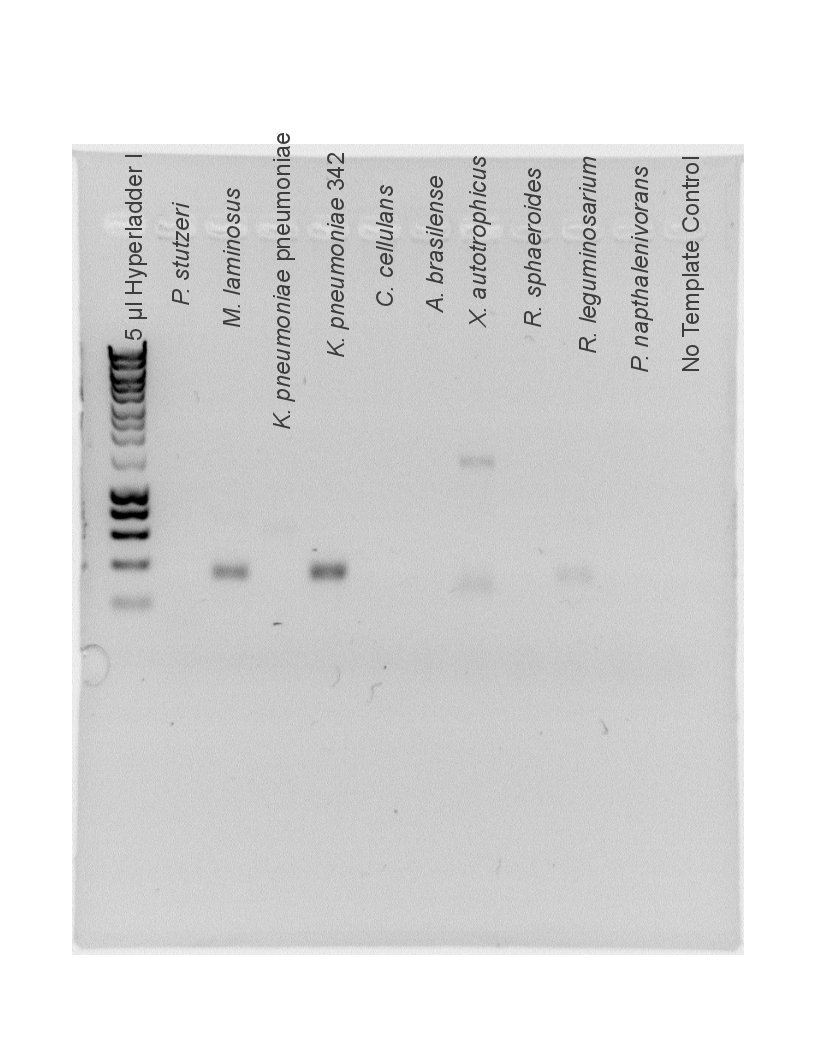

Supplement: Figure S10 — nifH2/R6 primer pair at 44°C annealing temperature. Gel image of PCR products generated using the primers indicated with a range of different DNA templates. Results are summarized and full strain names are reported in Table 6. The gel images have been inverted from black to white. (TIF) [file pone.0042149.s010.tif]

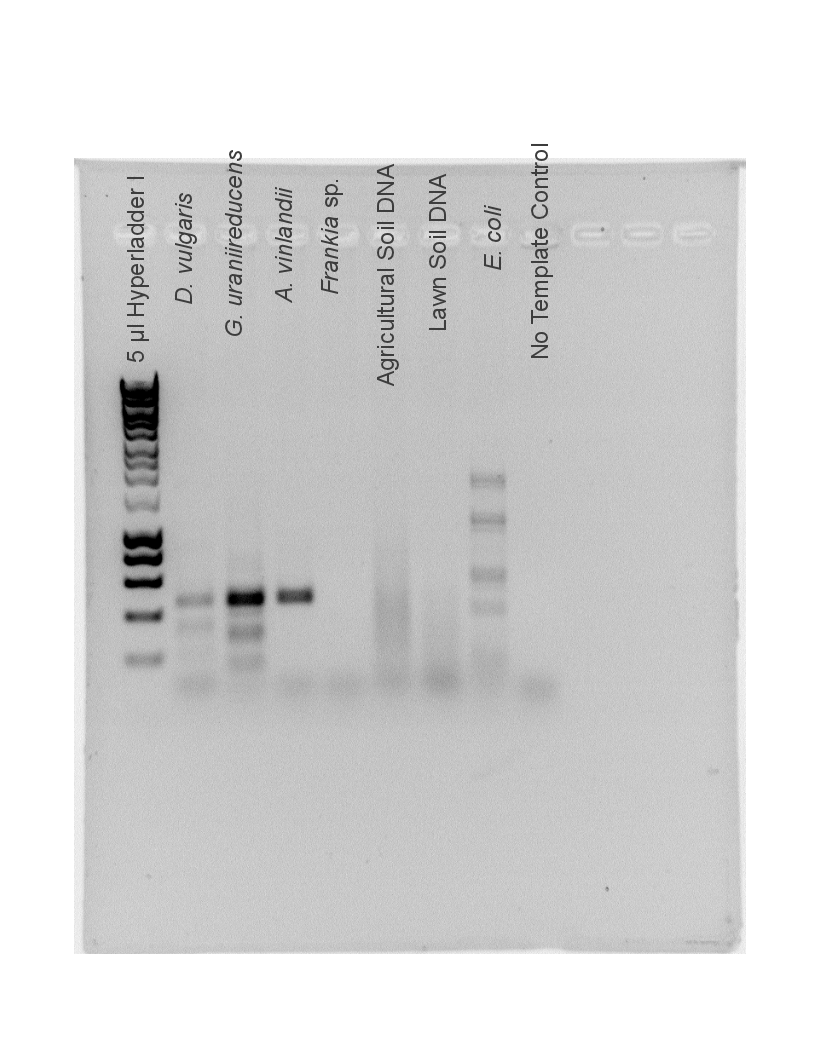

Supplement: Figure S11 — nH21f/nifH1 primer pair at 46°C annealing temperature. Gel image of PCR products generated using the primers indicated with a range of different DNA templates. Results are summarized and full strain names are reported in Table 6. The gel images have been inverted from black to white. (TIF) [file pone.0042149.s011.tif]

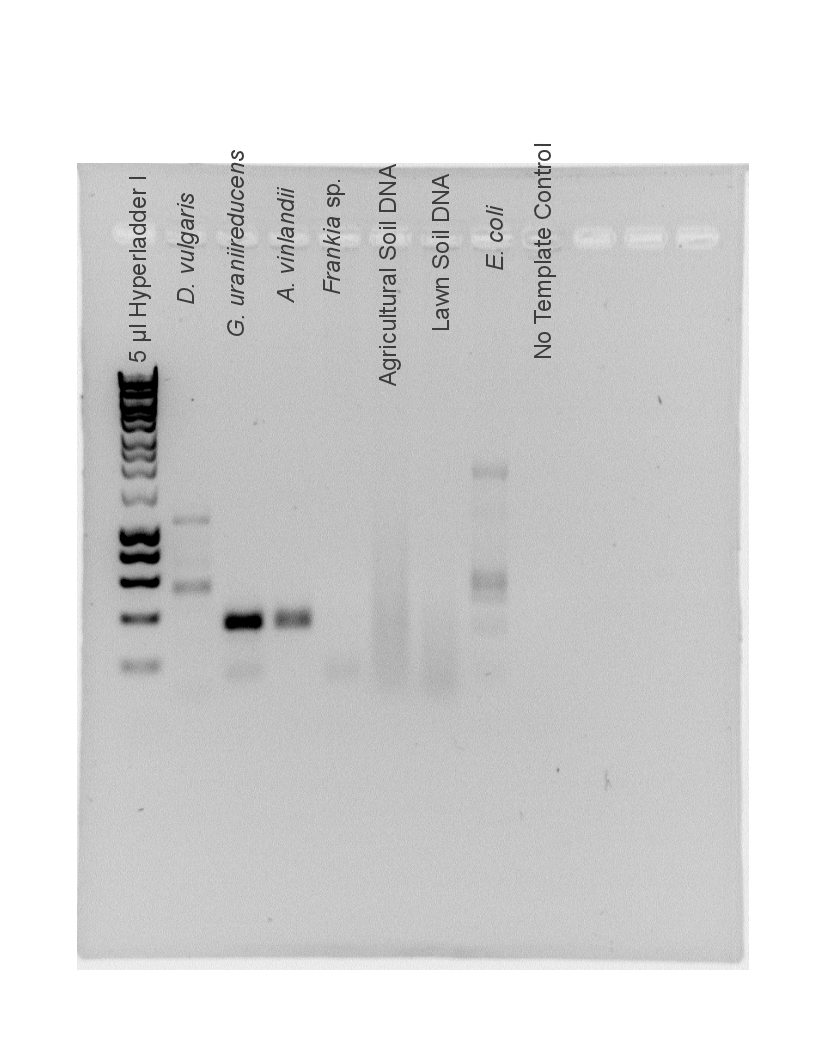

Supplement: Figure S12 — nifH1/nifH2 primer pair at 46°C annealing temperature. Gel image of PCR products generated using the primers indicated with a range of different DNA templates. Results are summarized and full strain names are reported in Table 6. The gel images have been inverted from black to white. (TIF) [file pone.0042149.s012.tif]

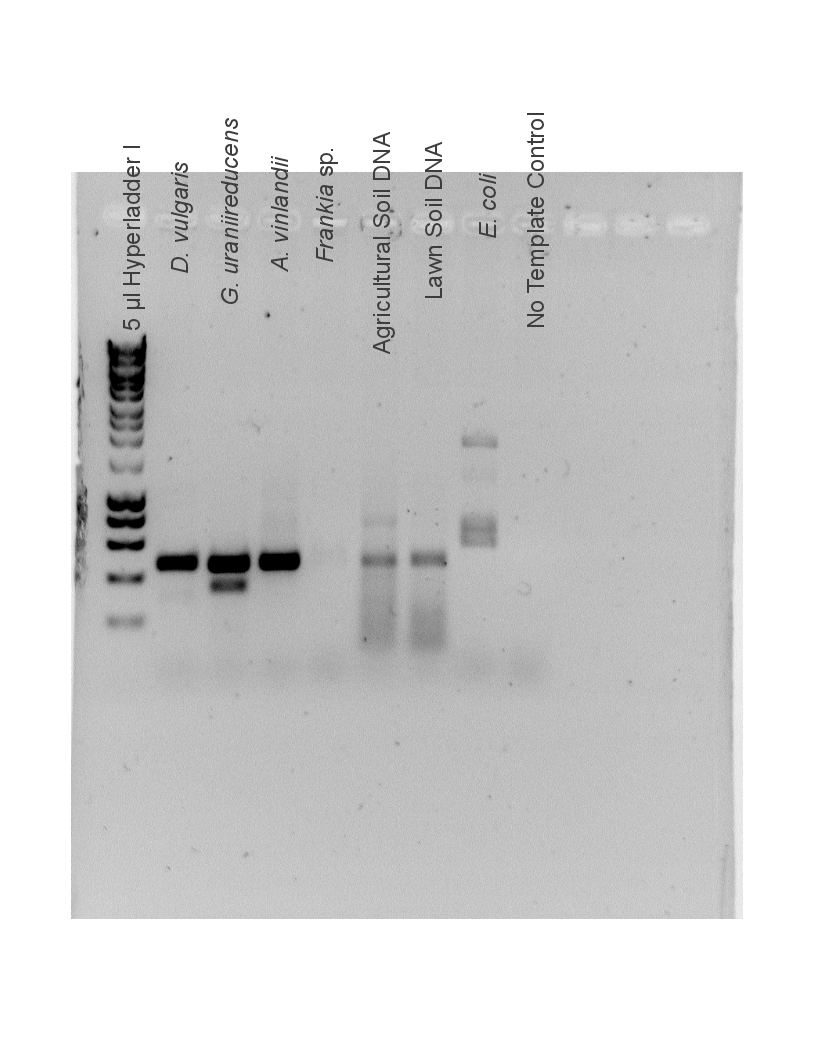

Supplement: Figure S13 — Ueda19f/univ463r primer pair at 46°C annealing temperature. Gel image of PCR products generated using the primers indicated with a range of different DNA templates. Results are summarized and full strain names are reported in Table 6. The gel images have been inverted from black to white. (TIF) [file pone.0042149.s013.tif]

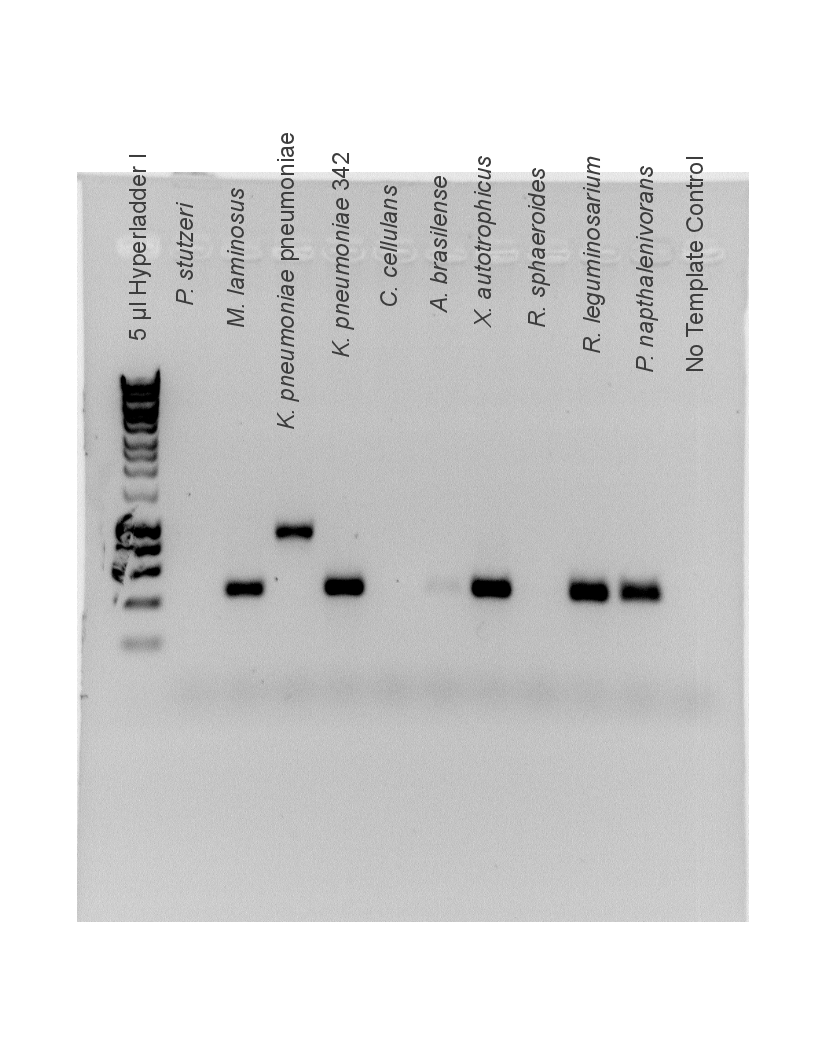

Supplement: Figure S14 — Ueda19f/univ463r primer pair at 46°C annealing temperature. Gel image of PCR products generated using the primers indicated with a range of different DNA templates. Results are summarized and full strain names are reported in Table 6. The gel images have been inverted from black to white. (TIF) [file pone.0042149.s014.tif]

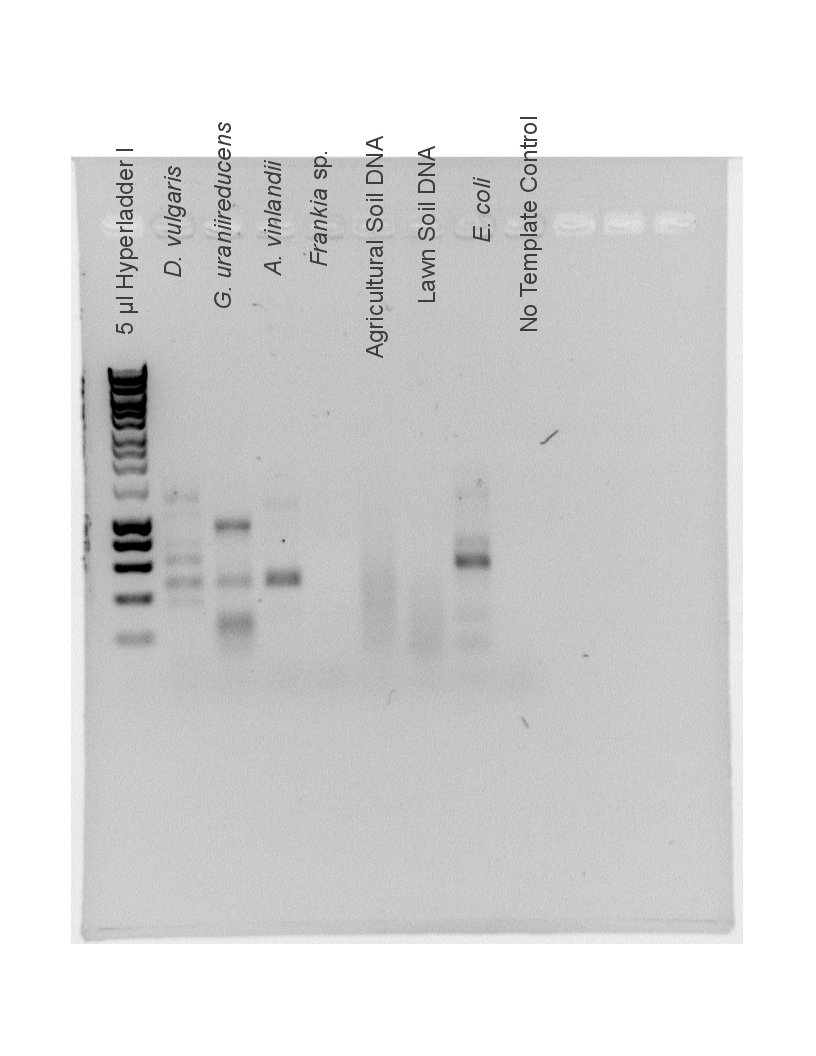

Supplement: Figure S15 — nifH3/nH21f primer pair at 41°C annealing temperature. Gel image of PCR products generated using the primers indicated with a range of different DNA templates. Results are summarized and full strain names are reported in Table 6. The gel images have been inverted from black to white. (TIF) [file pone.0042149.s015.tif]
